# CD47–SIRPα as a Therapeutic Target in Sarcoma: A Systematic Review of Expression, Preclinical, and Clinical Evidence

**DOI:** 10.3390/biomedicines14102187

**Published:** 2026-09-27

**Authors:** Misu Xiao, Cheng Guo, Quanjun Yang

**Affiliations:** Department of Pharmacy, Shanghai Sixth People’s Hospital, Shanghai Jiao Tong University School of Medicine, Shanghai 200233, China; xiaomisu@sjtu.edu.cn (M.X.);

**Keywords:** CD47, SIRPα, sarcoma, innate immune checkpoint, macrophage phagocytosis, “don’t eat me” signal, tumor-associated macrophage

## Abstract

**Background/Objectives:** Sarcoma comprises mesenchymal malignancies for which PD-1/PD-L1 checkpoint inhibitors have produced few durable responses. The CD47–SIRPα innate checkpoint suppresses macrophage phagocytosis through a “don’t eat me” signal and is the most clinically advanced myeloid checkpoint, but its evidence base in sarcoma has not been systematically assembled or appraised. **Methods:** A PRISMA 2020-compliant systematic review (PROSPERO CRD420261452040) was undertaken; three bibliographic databases and three trial registers, without language restriction, were searched on 14 September 2026. Expression, preclinical and clinical studies were appraised and graded separately. Synthesis followed SWiM guidelines and no pooled effect estimate was calculated. **Results:** Of 391 records identified, 50 studies met the eligibility criteria as separate analysis units (45 journal articles, 4 conference abstracts, 1 registry record), appraised across expression (20), preclinical (38) and clinical (18, including 2 interventional trials) domains. CD47 expression was subtype-dependent and bimodal, consistent in chordoma and angiosarcoma, frequently negative in leiomyosarcoma and Ewing sarcoma, and contested in undifferentiated pleomorphic sarcoma; reported positivity in osteosarcoma varied six-fold between cohorts, and the assay was often incompletely described. In preclinical models, CD47 blockade consistently increased phagocytosis and combination regimens outperformed single-agent use, but few studies used patient-derived or humanized models. Only two interventional trials enrolled sarcoma patients; both were single-arm, one was terminated without posted results, and nine of ten pharmacological strategies had no sarcoma-specific clinical data. Certainty was low for expression and patient-cohort evidence, and very low for preclinical and interventional evidence. **Conclusions:** The CD47–SIRPα axis is biologically well characterized and expressed in a definable subset of sarcomas, but has not been tested in these tumors in a design capable of detecting a treatment effect. Four of the fifty records were conference abstracts, which carry a higher risk of reporting bias and non-publication. The rate-limiting step lies in the assay and the trial architecture rather than in the biology: a harmonized immunohistochemical standard and a biomarker-selected trial with an internal comparator are prerequisites for an informative next study.

## 1. Introduction

Sarcomas encompass over 100 histological subtypes of mesenchymal origin, and they account for a small but disproportionate share of cancer-related mortality in adolescents and young adults [1]. For advanced disease, first-line treatment remains anthracycline-based chemotherapy, and median overall survival rarely exceeds 12–18 months [1]. Immune checkpoint inhibitors directed at PD-1 and PD-L1 have produced objective response rates of only about 5–20% in unselected sarcoma populations across the Alliance A091401 and SARC028 trials, and follow-up analyses have shown that durable responses are uncommon and concentrated in biomarker-defined subgroups [2,3,4,5]. Sarcoma is therefore a disease in which classical adaptive checkpoint blockade has largely failed, and in which alternative immunological targets warrant systematic evaluation. Notably, myeloid-directed therapy is not unprecedented in this disease: the antitumor activity of trabectedin in soft-tissue sarcoma is attributable in part to its effect on tumor-associated macrophages [6], which indicates that macrophages are not merely a bystander population in sarcoma.

The CD47–signal regulatory protein alpha (SIRPα) axis constitutes a critical innate immune checkpoint, a term used throughout this review to encompass the broader category of myeloid-regulatory interactions that include, but are not limited to, phagocytosis checkpoint mechanisms. CD47, a transmembrane immunoglobulin superfamily protein, is ubiquitously expressed but upregulated on cancer cells; its interaction with SIRPα on macrophages suppresses phagocytosis via a “don’t eat me” signal [7,8]. Because phagocytosis checkpoints act upstream of adaptive immunity, they represent a mechanistically distinct route to immune activation in tumors that are poorly responsive to PD-1-directed therapy, and CD47–SIRPα is the most clinically advanced of these pathways [9,10,11]. Beyond CD47, an expanding set of phagocytosis checkpoints is being catalogued—among them CD24, PD-L1, MHC class I and STC-1—against which CD47–SIRPα remains the most thoroughly studied and the most advanced in clinical translation [9]. Therapeutic blockade is achievable through several formats—anti-CD47 monoclonal antibodies, SIRPα-Fc fusion proteins, and CD47-directed bispecific antibodies—with important pharmacological differences between them. In particular, the antitumor activity of anti-CD47 antibodies depends on Fc–FcγR engagement, so antibody design determines whether blockade can generate a phagocytic signal or merely occlude the ligand: an Fc-silent format—such as the IgG4 backbone of magrolimab—cannot engage FcγR-mediated antibody-dependent cellular phagocytosis (ADCP) and rests on SIRPα occupancy alone, whereas Fc-active isotypes (IgG1, engineered IgG2) both block the axis and recruit FcγR-mediated effector functions [12]. A second and recurring constraint is that CD47 is expressed on erythrocytes and hematopoietic cells, creating a large peripheral antigen sink that limits tumor exposure and produces on-target hematological toxicity; this is the principal reason that the first-generation agent magrolimab was discontinued after its phase 3 ENHANCE program did not meet its endpoints [13]. Because its Fc-silent IgG4 design precludes FcγR-mediated effector recruitment, this format relies on SIRPα occupancy alone [12]—one proposed contributor to the limited single-agent activity of this class. The complementary “eat me” arm of the mechanism is supplied by tumor-cell death itself: certain cytotoxic agents, doxorubicin among them, induce surface exposure of calreticulin, which is required for phagocytic recognition of dying cells [14].

Development has since moved along three routes: Fc-active SIRPα-Fc fusion proteins that act as decoy ligands with an intact IgG1 Fc; CD47-directed bispecific antibodies engineered to restrict blockade to tumor cells—for example through PD-L1 guidance, which spares erythrocytes; and combinations in which a second agent supplies the pro-phagocytic signal [10,11]. Of these, only SIRPα-Fc fusion combined with chemotherapy has been evaluated in sarcoma [15]; the bispecific format has no sarcoma-specific data at all. The bispecific format is of interest because it couples innate and adaptive checkpoint blockade within a single molecule and rests on an affinity differential rather than on the CD47 arm alone: by engineering higher affinity for PD-L1 than for CD47, these constructs exploit the avidity effect of bispecific binding, so that strong PD-L1 engagement anchors the molecule to tumor cells while the low-affinity CD47 arm contributes to blockade only in the local tumor microenvironment, reducing systemic CD47 engagement.

Sarcoma is a plausible setting for this axis, but the supporting evidence has not been assembled systematically. Existing reviews of CD47-directed therapy have addressed the pathway across oncology as a whole [10,11,16,17]. What has been missing is an assessment that (i) treats CD47 expression, preclinical pharmacology, and clinical evidence as separately appraised domains rather than a single body of evidence; (ii) maps the evidence to individual sarcoma subtypes, because expression differs sharply between them; and (iii) is explicit about how much of the clinical literature is sarcoma-specific rather than extrapolated from other tumor types. Each of these distinctions changes the conclusions that can reasonably be drawn.

Despite these converging lines of evidence, a systematic synthesis of CD47-directed therapeutic strategies in sarcoma has been lacking. This review aims to critically evaluate whether the current evidence base, from expression profiling through clinical trials, is sufficient to support the initiation of rational, biomarker-driven clinical investigations of CD47–SIRPα-targeted therapies in sarcoma, and to identify the critical knowledge gaps that must be addressed. The certainty of the evidence is graded separately for each domain. Two design decisions follow from the published data and are stated here because they shape the interpretation throughout. Because the included studies span designs of fundamentally different evidentiary weight, findings are reported by domain and are not combined into a single summary estimate; no pooled effect size is presented. Because expression is subtype-dependent, conclusions are reported at subtype level wherever the data permit.

This review encompasses adult-type STS (UPS, DDLPS/WDLPS, LMS, MFS, synovial sarcoma) and bone sarcomas (osteosarcoma, Ewing sarcoma). Myeloid sarcoma, a hematological malignancy with extramedullary presentation that is pathobiologically distinct from mesenchymal sarcoma, appears in the expression data for completeness (Section 3.2.1) but is outside the therapeutic scope. Figure 1 summarizes the CD47–SIRPα mechanism and the pharmacological strategies discussed in this review.

## 2. Materials and Methods

### 2.1. Protocol and Registration

This systematic review was conducted in accordance with a protocol registered prospectively in PROSPERO (CRD420261452040). The record was first published on 15 July 2026; the version that defines the search strategy as executed in this review (v3.0) was published on 12 September 2026, i.e., before the searches were run (Section 2.2). Reporting follows the PRISMA (Preferred Reporting Items for Systematic Reviews and Meta-Analyses) 2020 statement [18] (Table S11) and the PRISMA-S extension for search reporting. The amendments are described in the record’s revision note and the searches executed under the amended protocol are given in full in Table S1. No protocol deviation affecting the eligibility criteria or the synthesis method was introduced after registration.

### 2.2. Search Strategy

Databases and registers. Three bibliographic databases—Embase (Elsevier, Amsterdam, The Netherlands), Web of Science Core Collection (SCI-EXPANDED, SSCI, ESCI and CPCI-S) and PubMed/MEDLINE—and three trial registers—ClinicalTrials.gov, the WHO International Clinical Trials Registry Platform (ICTRP) and the EU registers (EU CTR legacy and CTIS)—were searched. Records were restricted by publication date from 1 January 2014 to 30 September 2026 (day-level granularity, i.e.,/pd). No language, geographical, or publication-status restriction was applied—as registered, and stated explicitly as “There are no language restrictions” in the PROSPERO record. The final searches were run on 14 September 2026. Conference abstracts indexed in Embase and Web of Science were additionally eligible as a source of study reports. The number of records retrieved from each source is shown in Figure 2; the verbatim search strings are given in Table S1.

Search terms. Three concept blocks were combined with AND: (i) disease—sarcoma and histology-specific terms (osteosarcoma, Ewing sarcoma, leiomyosarcoma, liposarcoma, rhabdomyosarcoma, chondrosarcoma, synovial sarcoma, undifferentiated pleomorphic sarcoma, gastrointestinal stromal tumor, and others); (ii) target—CD47/SIRPα axis terms (CD47, SIRPα, signal regulatory protein alpha), expanded with drug names (magrolimab, Hu5F9-G4, ontorpacept, TTI-621/TTI-622, evorpacept, ALX148, lemzoparlimab, IBI188, TQB2928, HCB101, IMM2520, PF-07257876, SGN-CD47M); (iii) modality—immunotherapy, antibody, bispecific, Fc fusion, immunohistochemistry, preclinical, clinical trial, pharmacokinetics. No study-design filter was applied. Full search strings, verbatim as executed, are provided in Table S1.

### 2.3. Eligibility Criteria

Studies were eligible if they met all of the following criteria:

Population. Studies reporting on human sarcoma of any histological subtype arising from bone or soft tissue. Studies focused exclusively on non-sarcomatous malignancies, or investigating CD47–SIRPα biology solely in hematological cancers or carcinomas, were excluded. Where CD47 or SIRPα was measured only as part of a pan-cancer panel, studies were excluded unless sarcoma data could be extracted independently. For preclinical work, models of non-sarcoma tumors and in vitro systems lacking any sarcoma-derived component were excluded.

Intervention/exposure. Any agent engaging the CD47–SIRPα axis (CD47- or SIRPα-blocking monoclonal antibodies, SIRPα-Fc fusion proteins, CD47/SIRPα bispecific engagers, and combinations), or, for expression studies, quantification of CD47 or SIRPα in sarcoma tissue. Interventions not engaging the axis (e.g., agents targeting PD-L1, TIGIT or TIM-3 in isolation) were excluded.

Terminology. Throughout this review, “bispecific antibody” is used in its strict molecular sense: a single molecule that targets two distinct antigens through independent binding domains, as in the CD47×PD-L1 bispecifics PF-07257876 and IMM2520. SIRPα-Fc fusion proteins such as HCB101 instead carry two identical SIRPα domains directed at the same antigen (CD47) and are therefore classified as bivalent fusion proteins, not bispecific antibodies. This distinction is applied consistently.

Comparator. Any study-defined comparator accompanying a CD47–SIRPα-targeted intervention; for expression studies, the implicit comparator was sarcoma subtype or non-tumor tissue.

Outcomes. For expression studies, the proportion of sarcoma specimens or patients with membrane or cytoplasmic CD47/SIRPα expression, together with associations with clinicopathological variables; for preclinical studies, functional outcomes (phagocytosis, tumor growth, metastasis, survival) in sarcoma models; for clinical studies, response, survival, and safety outcomes.

Study design. Original research addressing any of the three evidence domains (expression profiling, preclinical pharmacology, early-phase clinical studies) was eligible. Conference abstracts reporting extractable outcome or expression data were eligible. Standalone systematic reviews, meta-analyses and practice guidelines were excluded from the primary synthesis but may be cited narratively. Purely computational or in silico studies lacking experimental validation were excluded. Case reports describing single patients without quantifiable population-level data were excluded from the expression and preclinical syntheses but may be noted narratively for safety signal triangulation.

### 2.4. Study Selection and Data Extraction

Records were deduplicated before screening. Titles and abstracts were screened against the eligibility criteria; full texts of potentially eligible reports were then assessed. Screening and full-text assessment were performed in duplicate by two independent reviewers (M.X. and Q.Y.); disagreements were resolved by discussion between the two reviewers. Eligibility judgements were recorded at the level of the individual record in a machine-readable adjudication file, so that every decision is traceable to its supporting evidence.

Data were extracted into standardized forms and comprised: bibliographic identifiers (DOI, PMID, trial registration number); sarcoma subtype(s); evidence domain; study design; specimen source and sample size; CD47/SIRPα assay characteristics (antibody clone, scoring method, positivity threshold); intervention and comparator; outcome definitions and results; and funding and conflict-of-interest statements. Where a study reported CD47 positivity, the numerator and denominator were extracted explicitly so that proportions could be recomputed. When multiple reports of the same study were identified, the most complete report was retained and the others were excluded to avoid double counting.

### 2.5. Quality and Certainty Assessment

Risk of bias was assessed with instruments matched to each evidence domain. Risk of bias was assessed with a modified Newcastle–Ottawa Scale (six items: cohort representativeness, sample size, assay description, definition of the positivity threshold, control for confounders, and adequacy of outcome statistics) applied to expression-profiling and patient-cohort studies. For preclinical studies, methodological quality and reporting were appraised with a SYRCLE-informed checklist covering model type, in vivo validation, randomization, blinding, sample-size justification, and pre-specification of dose and endpoint. Because model fidelity is the principal determinant of translational relevance, tumor origin (patient-derived, human cell line, or murine) and host immune status (immunocompetent versus immunodeficient) were recorded as two orthogonal dimensions rather than collapsed into a single score. The interventional trials were appraised for randomization, presence of a control arm, and availability of posted results. Items not reported in the source publication were recorded as not reported rather than scored as absent, so that reporting completeness could be summarized transparently as a proportion.

Assessments were completed independently by two reviewers (M.X. and Q.Y.), with disagreements resolved by discussion; additional information was not sought from investigators. Certainty of evidence was graded per domain using a GRADE-informed framework (risk of bias, indirectness, imprecision, inconsistency, and publication bias). Because the domains differ fundamentally in design and outcome metric, certainty is reported separately for each domain and is not aggregated into a single summary rating. Per-study assessments are provided in Table S2 (expression and patient-cohort studies), Table S3 (interventional trials) and Table S4 (preclinical studies); the domain-level basis for the certainty ratings is given in Table S5.

### 2.6. Synthesis Strategy

Because the included evidence spans expression profiling, preclinical pharmacology, and early-phase clinical studies that differ fundamentally in design, population, and outcome metric, data were combined through structured narrative synthesis following the SWiM (Synthesis Without Meta-analysis) reporting guideline [19]. No meta-analysis with pooled effect sizes was performed, and no summary estimate is presented in any figure or table; individual study estimates are displayed without pooling. Where studies reported proportions, Wilson score 95% confidence intervals were computed for descriptive display only.

Findings are organized by evidence domain. An evidence map was constructed to display the distribution of included studies across sarcoma subtypes and evidence domains, so that areas of absent or sparse evidence are made explicit. Heterogeneity across studies—in sarcoma subtype, assay, model system, and outcome definition—is described rather than statistically quantified, and is reported as a finding in its own right where it affects interpretability.

## 3. Results

### 3.1. Study Selection and Characteristics

Consistent with the synthesis plan set out in Section 2.6 (SWiM; no pooled estimate), findings are presented separately for each evidence domain rather than combined. The distribution of evidence across subtypes and domains is summarized as an evidence map in Figure 3. No case report met the eligibility criteria, so the narrative safety triangulation described in Section 2.3 was not required.

The searches identified 391 records, of which 130 were duplicates, leaving 261 records screened at title and abstract level. Full texts of 182 reports were sought and all were retrieved; 132 were subsequently excluded, most often because they were reviews or other non-primary publications (*n* = 51) or reported no CD47–SIRPα data (*n* = 31) (Table S6). A total of 50 studies met the eligibility criteria and were included as separate analysis units: 45 journal articles, 4 conference abstracts, and 1 registry record. Two further reports described the same studies as an included journal article and were therefore counted with their full report rather than separately (see Section Evidence Is Concentrated in a Single Subtype, below) (Figure 2).

The included studies span three evidence domains that differ fundamentally in design and evidentiary weight and should not be interpreted as a homogeneous evidence base: expression profiling (*n* = 20), preclinical studies (*n* = 38), and clinical or translational studies (*n* = 18) (2 interventional trials and 16 patient-cohort studies). Because a single study may contribute to more than one domain or address more than one sarcoma subtype, the domain-level counts exceed the number of included studies. The distribution of evidence across sarcoma subtypes and domains is shown in Figure 3; 19 of the 48 subtype × domain cells (40%) contain no evidence at all, so the map should be read as much for its gaps as for its contents.

Publication years ranged from 2014 to 2026, with more than half of all studies published in 2023 or later (Figure 3B), indicating a rapidly emerging field rather than a mature evidence base.

#### Evidence Is Concentrated in a Single Subtype

Osteosarcoma was addressed by 35 of the 50 studies (70%), and accounted for 30 of the preclinical studies. The remaining subtypes were represented by 5 or fewer studies each (Ewing sarcoma 5, undifferentiated pleomorphic sarcoma 4, liposarcoma 3, leiomyosarcoma 3, fibrosarcoma 2, two pan-sarcoma series, and 1 study each for chondrosarcoma, angiosarcoma, myxofibrosarcoma, myeloid sarcoma, and solitary fibrous tumor). Ten of twelve subtypes had no interventional trial evidence at all. This distribution is a property of the field rather than of the search, and it constrains every inference drawn below.

### 3.2. CD47 Expression Across Sarcoma Subtypes

#### 3.2.1. Expression Is Neither Universal nor Uniform

The largest available survey assessed 1242 sarcoma specimens spanning 24–27 histological types by immunohistochemistry, using clone B6H12 and scoring the percentage of positive tumor cells [20]. Of these specimens, 47.5% showed at least focal CD47 expression, and the distribution of scores was distinctly bimodal: most cores stained either 0% or ≥90% of tumor cells. A single summary statistic therefore misrepresents this distribution, and the statement that “CD47 is overexpressed in sarcoma” is not supportable at the level of the disease as a whole (Figure 4A,B).

The two largest subtype-resolved series disagree, and the disagreement must be read as part of the result. In the 1242-specimen survey, undifferentiated pleomorphic sarcoma was the clearest low-expresser subtype (82% of cases negative). In contrast, a 55-patient treatment-naïve series reported that more than 80% of high-grade undifferentiated pleomorphic sarcoma samples showed moderate-to-diffuse positivity, and a further study reported CD47 expression in 86.9% of a mixed soft-tissue sarcoma cohort and observed its most pronounced ex vivo response to anti-CD47 in an undifferentiated pleomorphic sarcoma [24,25]. The two smaller studies therefore point in the opposite direction to the largest one for the same subtype. Both cannot be correct as stated, and the difference cannot presently be attributed to biology rather than to sampling, scoring or assay, which is precisely why a harmonized standard (Section 4.3) is required before CD47 is used to select patients.

Three methodological explanations are more plausible than a biological one. First, the 1242-specimen survey used a tissue microarray (cores of <1 mm), whereas the 55-patient series scored whole FFPE sections; core sampling systematically under-represents heterogeneous staining and would bias the larger study towards negativity. Second, the smaller series was restricted to high-grade tumors, and reported a trend towards higher expression in grade 3 than in grades 1–2. Third, the two studies used non-comparable scoring systems (percentage of positive tumor cells versus an area-based 0–3+ scale), and the smaller series did not report its antibody clone. Whether undifferentiated pleomorphic sarcoma is a high- or a low-expresser subtype therefore cannot be resolved from the current literature.

A further caveat applies to this comparison. The 55-patient and 66-patient series were both recruited at the same surgical department in the same hospital over overlapping periods (2016–2021 and 2019–2021), so they may include overlapping patients; their agreement should therefore not be read as two fully independent confirmations.

Setting that conflict aside, the remaining subtype-level signals were more consistent (Table 1).

This heterogeneity is directly relevant to trial design: the same survey concluded that some of the most common sarcomas express CD47 infrequently, which would produce false-negative results when trials enroll undifferentiated soft-tissue sarcoma populations. Conversely, chordoma and angiosarcoma—rare subtypes that are poorly served by conventional therapy—emerged as the most consistent expressers.

#### 3.2.2. Reported Positivity in Osteosarcoma Varies Six-Fold Between Cohorts

Even within a single subtype, reported CD47 positivity diverged markedly (Figure 4C): in 81 tissue-microarray specimens stained with clone B6H12, 87.7% (95% CI 79–93) of osteosarcoma samples were CD47-positive [21], whereas two more recent cohorts reported 20.8% (95% CI 9–40) among 24 diagnostic biopsies [22] and 14.3% (95% CI 6–29) among 35 heavily pre-treated patients [23].

The interval for the tissue-microarray cohort does not overlap either of the two later cohorts, although those two overlap each other. Plausible contributors include specimen type (tissue microarray versus whole section), the positivity threshold applied, prior treatment, and the antibody clone and detection platform used. Consistent with this, appraisal of the included expression and cohort studies found that the assay was described in only 45% of studies and a positivity threshold defined in 85% (Section 3.5). Cross-study comparison of CD47 positivity rates is therefore not interpretable until assay and scoring are harmonized—a prerequisite for using CD47 as a biomarker.

#### 3.2.3. Only a Minority of Studies Report a Quantifiable CD47 Result

Of the 20 studies in the expression and patient-cohort domains, only 10 yielded a CD47 positivity figure with an identifiable source sentence (Table S7). Of the remaining 10, 3 contributed patient-level or biomarker data without reporting a positivity rate, and 7 reported no extractable CD47 quantification at all; throughout this domain, the antibody clone or detection platform was left unspecified in the majority of studies. This is not a limitation of data extraction: it reflects what the source publications report, and it is consistent with the appraisal in Section 3.5, where the assay was described in 45% of studies.

The practical consequence is that the evidence base cannot presently support a pooled or cross-study estimate of CD47 positivity in sarcoma even if such an analysis were intended—not because pooling is methodologically undesirable in principle, but because the underlying measurements are not commensurable. This is the empirical basis for the assay-harmonization recommendation in Section 4.3.

#### 3.2.4. The Prognostic Direction Is Inconsistent

The relationship between CD47 expression and outcome differed between reports. In the 1242-specimen survey, CD47 expression was an adverse prognostic factor in osteosarcoma but not in solitary fibrous tumor; in the same study, the adverse prognostic feature in synovial sarcoma and myxofibrosarcoma was the presence of SIRPα^+^ macrophages, not CD47 expression itself [20]. In contrast, in soft-tissue leiomyosarcoma high CD47 expression was associated with improved survival (HR 0.76, 95% CI 0.08–0.88, *p* = 0.021) [26]. In myeloid sarcoma, CD47 expression was reported as a favorable prognostic factor [27], although in that study it was not an independent predictor in multivariable analysis and no significant difference was observed in progression-free survival. These estimates derive from small, single-center cohorts—the leiomyosarcoma result rests on 55 patients, and its confidence interval is wide—so the reported discrepancy between subtypes should be interpreted with caution rather than taken as established biology.

### 3.3. Preclinical Evidence

#### 3.3.1. Overview and Scope

A total of 38 preclinical studies were included. Table S8 tabulates, for each study, the sarcoma model, model fidelity (with tumor origin and host immune status reported as separate dimensions), the combination partner, and the key functional finding.

Combination is the norm rather than the exception. Of the 38 preclinical studies, 24 (63%) tested CD47–SIRPα blockade alongside at least one partner modality, whereas 14 (37%) assessed the agent as monotherapy or examined CD47 biology without a therapeutic intervention. Partners fell into four broad groups: delivery and formulation strategies—nanoparticle, liposomal, hydrogel or framework-based carriers (*n* = 11); stimuli that provoke apoptosis or immunogenic cell death, chiefly radiotherapy, sonodynamic or photothermal therapy and calreticulin exposure (*n* = 6 and *n* = 8, respectively); conventional chemotherapy (*n* = 4); and a second immune checkpoint (*n* = 3; Table S8). Smaller numbers paired blockade with engineered cellular therapy, a targeted kinase or STAT3 inhibitor, oncolytic virotherapy, or an innate immune agonist (*n* = 2, 2, 2 and 1, respectively). Table S8 gives the model system, combination partner and key functional finding for every study. Because a single study may use more than one partner, these categories overlap and do not sum to 38.

Two features of this distribution are worth noting. The predominance of nanocarrier-based formulations reflects a delivery problem rather than a mechanistic one: CD47 is widely expressed on normal tissues, so tumor-selective delivery is a central engineering challenge for this target (Section 3.3.2). Formal combination analyses were rarely reported, however, even among studies whose titles or abstracts describe the combination as synergistic—a point developed in Section 3.3.2 and relevant to the appraisal in Section 3.5.

#### 3.3.2. What the Preclinical Literature Consistently Shows—And What It Does Not

Across studies, three findings recur:

CD47 blockade increases macrophage-mediated phagocytosis of sarcoma cells in vitro, including in osteosarcoma, Ewing sarcoma and undifferentiated pleomorphic sarcoma models (representative studies: [21,22,25,28]).

Combination is consistently superior to single-agent CD47 blockade, whether the partner is chemotherapy, radiotherapy, a second innate or adaptive checkpoint, an oncolytic virus, or a nanocarrier (representative studies: [29,30,31,32]). Throughout this review, “superior” is defined strictly as greater antitumor efficacy—tumor growth inhibition in vivo and, where reported, survival—relative to single-agent blockade under the same experimental conditions; it is a statement about efficacy endpoints only, and not about immune activation generally. This should not be read as evidence that adding blockade is always beneficial. One included study—reported in both a conference abstract and its subsequent full paper, and counted once—found combination results that were flat: in freshly resected soft-tissue sarcoma tissue, all samples cultured with both anti-PD-1 and anti-CD47 showed a reduction in cytokine secretion relative to single-agent culture [25]; co-administration of anti-PD-1 and anti-CD47 likewise did not further increase pro-inflammatory cytokine production over either agent alone [25]. Neither observation establishes antagonism in vivo, but both indicate that the superiority claimed here is endpoint-specific: it holds for antitumor efficacy as defined above, and does not extend to cytokine secretion or other immune-activation readouts.

Antitumor activity is most convincing in osteosarcoma models, which also constitute the majority of the preclinical literature (30 of 38 studies).

Three limitations qualify these findings, all of which are measurable rather than speculative:

Model fidelity. Only 9 of 38 appraised preclinical studies (24%) used patient-derived or humanized models; 17 (45%) used human sarcoma cell lines implanted in immunodeficient hosts—models that carry human tumor cells but not a human immune system. Because CD47–SIRPα blockade acts through macrophage–T-cell crosstalk, reliance on immunodeficient xenografts is a substantive constraint on translational inference, not a formality (Section 3.5).

Reporting of methodological safeguards. Randomization was reported in 42%, blinding in 32%, sample-size justification in 13%, and pre-specification of dose and endpoint in 24%. These figures describe reporting completeness; whether the safeguards were absent or merely unreported cannot be determined from the publications.

Absence of a formal combination analysis. Several studies describe CD47 blockade as synergistic with a partner agent. Where the underlying studies did not perform a formal interaction analysis (Bliss independence, Loewe additivity, or a combination index), such claims rest on the combination outperforming control, which establishes additivity at best. This review therefore uses “combined with” or “enhanced the effect of” unless a formal synergy metric was reported.

### 3.4. Clinical and Translational Evidence

#### 3.4.1. Interventional Trials (*n* = 2)

Only two interventional trials enrolling sarcoma patients were identified worldwide: NCT04751383 (magrolimab plus dinutuximab) [33] and NCT04996004 (ontorpacept plus doxorubicin) [15] (Table S9; Figure 5A).

Both were single-arm and non-randomized. For NCT04751383, termination was attributed to drug supply issues and no results were posted, so no outcome data could be extracted. For NCT04996004, the reported objective response rate in leiomyosarcoma cannot be attributed to CD47–SIRPα blockade. The trial had no control arm, and the source publication itself notes that response to doxorubicin monotherapy in soft-tissue sarcoma is approximately 13–23%, with a median progression-free survival of 6–7 months [38]—a range that encompasses the reported combination result. The observed response is therefore best described as that of the combination, and does not establish an incremental contribution from CD47–SIRPα blockade. Safety findings from this trial are informative, however: grade ≥3 neutropenia occurred in 66% of patients and grade 3/4 thrombocytopenia was more frequent than anticipated at the 2.0 mg/kg dose level, consistent with the on-target hematological toxicity that has constrained this drug class.

#### 3.4.2. Patient Cohorts and Translational Studies (*n* = 16)

Sixteen studies used human sarcoma specimens or patients to relate CD47 or SIRPα to clinical variables; Table S10 lists each study, its cohort and its outcome measure. Collectively these are appreciably deeper than the interventional evidence: sample sizes ranged from 8 to 1242 (median 66.5; 14 of the 16 studies reported a sample size), and the studies cover tissue-based immunohistochemistry, serum and plasma macrophage biomarkers, imaging, and transcriptomic profiling (Figure 5).

Three observations follow. The CD47 axis is measurable in patients with assays already available in pathology practice. The associations point in different directions across subtypes (Section 3.2.3), which undermines the assumption that CD47 is uniformly a negative prognostic marker. Several cohorts, moreover, measured macrophage biology rather than CD47 itself—for example serum sSIRPα and sCD163 predicted mortality in a 152-patient soft-tissue sarcoma cohort [34]—indicating that the axis can be interrogated through soluble biomarkers, a route not yet exploited in trial design.

#### 3.4.3. The Interventional—Observational Gap

The asymmetry between these two subsections is the central clinical finding of this review and is shown in Figure 5: observational evidence has accumulated continuously since 2015 and covers six subtypes, whereas interventional evidence consists of two early-phase trials concentrated in 2021 and 2026. Of the ten pharmacological strategies targeting this axis that were identified across the included studies (Table 2), only one—combination with conventional chemotherapy—has sarcoma-specific clinical data, and even there the contribution of CD47–SIRPα blockade cannot be isolated. The remaining nine strategies, including CD47 blockade as monotherapy, have been evaluated only preclinically.

The rate-limiting step in this field is the near-absence of prospective interventional studies rather than any shortage of sarcoma tissue data. This reframes an apparent weakness—a thin clinical evidence base—as a specific and measurable gap. It is also the factual basis for the trial-design recommendations in Section 4.3.

### 3.5. Risk of Bias and Certainty of Evidence

Risk of bias was assessed per evidence domain (Tables S2–S4; the domain-level basis for the certainty ratings is given in Table S5; methods in Section 2.5). Across expression and patient-cohort studies, the weakest items were control for confounders (reported in 45%) and description of the assay (45%), with a median of 4 of 6 items met. Among preclinical studies, the most frequently unreported items were sample-size justification (13%), pre-specified dose and endpoint (24%), blinding (32%) and randomization (42%). Certainty of evidence was graded separately for each domain (Table 3):

Certainty is reported per domain and is not aggregated, because the domains differ fundamentally in design and outcome metric. It should be noted that these ratings are not a matter of editorial conservatism: they follow directly from the measured reporting frequencies and from the model composition of the underlying literature.

## 4. Discussion

### 4.1. Principal Findings

This review synthesizes 50 studies and maps the CD47–SIRPα evidence base in sarcoma across expression, preclinical and clinical domains simultaneously; we are not aware of a previous synthesis that does so at subtype resolution. Four findings are central.

First, CD47 expression in sarcoma is subtype-dependent rather than uniformly high. In the largest available survey, 47.5% of 1242 specimens showed at least focal expression, and the distribution was bimodal: most specimens were either entirely negative or showed ≥90% positive tumor cells. Chordoma and angiosarcoma expressed CD47 consistently. For the commoner subtypes the evidence conflicts: the largest series found undifferentiated pleomorphic sarcoma, leiomyosarcoma and Ewing sarcoma to be frequently negative, whereas a 55-patient series reported moderate-to-diffuse positivity in more than 80% of high-grade cases and a further study observed its strongest ex vivo anti-CD47 response in an undifferentiated pleomorphic sarcoma. We therefore report this subtype as contested rather than as a low-expresser, because the smaller reports point the other way, and the discrepancy is at least as likely to reflect sampling and scoring as biology. The statement that “CD47 is overexpressed in sarcoma”, which has been carried forward in the introduction of a substantial number of preclinical papers, is therefore not supportable at the level of the disease as a whole.

Second, even within one subtype the measurement is not reproducible. Three osteosarcoma cohorts reported CD47 positivity of 87.7%, 20.8% and 14.3%, the interval for the first cohort does not overlap either of the other two. Because the assay was described in only 45% of studies, the discrepancy cannot be attributed to any single methodological factor, but specimen type, positivity threshold and prior treatment are all plausible contributors. This is a measurement problem before it is a biological one, and it has a direct consequence: cross-study comparison of CD47 positivity is not interpretable in the current literature.

Third, the preclinical literature is large, internally consistent in direction, and limited by its models. Across 38 preclinical studies, CD47 blockade consistently increased macrophage-mediated phagocytosis, and combination with a partner modality was consistently superior to single-agent blockade (24 of 38 studies; Table S8). However, only 9 of 38 appraised studies (24%) used patient-derived or humanized models, whereas 17 (45%) used human sarcoma cell lines in immunodeficient hosts. Because CD47–SIRPα blockade is proposed to act through macrophage–T-cell crosstalk, the latter models are, by construction, unable to test the mechanism being invoked—a constraint that is structural rather than incidental.

Fourth, and most consequentially, the clinical evidence is sparse and narrowly distributed across strategies. Only two interventional trials have enrolled sarcoma patients, both single-arm and one terminated without posted results. Across the ten pharmacological strategies identified (Table 2), only one has sarcoma-specific clinical data: combination with chemotherapy. Even there, the contribution of CD47 blockade cannot be isolated. For the remaining nine strategies, no sarcoma-specific clinical evaluation has been reported; these include CD47 blockade as monotherapy, nanocarrier delivery, radiotherapy combination, and co-targeting of a second innate or adaptive checkpoint.

### 4.2. Why the Preclinical-to-Clinical Transition Has Stalled

The usual explanation for the slow translation of CD47-directed therapy is on-target hematological toxicity, and this review supports it. In the one sarcoma trial with reported safety data, grade ≥3 neutropenia occurred in 66% of patients, and grade 3/4 thrombocytopenia was more frequent than anticipated at the higher dose level. However, the present analysis identifies a second and less-discussed constraint that is specific to sarcoma.

For the commonest sarcoma subtypes the largest survey reports expression in only a minority of cases—leiomyosarcoma and Ewing sarcoma consistently so, and undifferentiated pleomorphic sarcoma contested (Section 4.1)—and expression is bimodal rather than graded. Trials that enroll biomarker-unselected soft-tissue sarcoma populations will therefore dilute any treatment effect toward the null. This is not a hypothetical concern: it follows directly from the subtype distribution in Figure 4 and was anticipated by the largest expression survey, which explicitly warned that pooling sarcoma types may produce false-negative results. A biomarker-unselected sarcoma trial of CD47 blockade is therefore not a neutral first step; it is an experiment that the available expression data predict will be uninformative.

The converse constraint applies to biomarker-selected designs, and undifferentiated pleomorphic sarcoma is its clearest illustration. The largest survey found this subtype among the lowest CD47 expressers, whereas smaller series report more than 80% positivity in high-grade cases (Section 4.1); with the assay described in only a minority of studies, this discrepancy cannot be resolved from the existing literature. Until a harmonized CD47 assay exists, any CD47-based patient-selection threshold—for this subtype above all—is arbitrary: the positivity rate a trial would select on is a property of the chosen antibody clone, platform and scoring cut-off rather than of the tumor, so two trials applying nominally “the same” biomarker criterion could enroll biologically different populations and reach opposite conclusions. Biomarker-guided selection remains the correct architecture for this axis, but for undifferentiated pleomorphic sarcoma it is not currently executable; the harmonized immunohistochemical standard recommended in Section 4.3 is the precondition that would make it non-arbitrary.

A third element is pharmacological rather than disease-specific. CD47 is expressed on normal hematopoietic cells and on erythrocytes, so systemic blockade produces a predictable antigen-sink effect: dose is consumed by normal tissue before it reaches tumor, and the same binding that produces antitumor phagocytosis produces anemia, thrombocytopenia and neutropenia, reflecting the physiological role of CD47 in regulating red-cell clearance [39]. This is why 11 of 38 preclinical studies in this review used a nanocarrier, liposomal, hydrogel or framework-based delivery strategy rather than the unmodified antibody—the delivery problem, not the target, has driven much of the recent engineering effort. Any sarcoma program will inherit this constraint, and it is the principal reason that a well-tolerated tumor-selective format is likely to be a precondition for testing the axis in a rare-disease population where the therapeutic index must be favorable. Formats engineered specifically to minimize erythrocyte and platelet binding have already been described [40], so this constraint is addressable rather than intrinsic.

The preclinical model composition compounds this. If the mechanism of action depends on macrophage–T-cell interaction, then the studies that rely on human sarcoma cell lines in immunodeficient hosts—17 of 38 (45%)—cannot provide evidence for or against it. This matters all the more because tumor-associated macrophages are themselves functionally heterogeneous, with distinct subpopulations exerting opposing effects [41], so “macrophage activation” is not a single measurable outcome. The preclinical literature is best read not as a body of efficacy evidence awaiting clinical confirmation, but as a set of mechanistic proofs of concept in systems that are not designed to test the proposed mechanism in a human-relevant immune context.

### 4.3. Recommendations: What Would Make the Next Study Informative

Because the limitations identified here are measurable and specific, they translate into concrete recommendations rather than general calls for more research.

For expression studies and biomarker development. Reporting should specify (i) the antibody clone and detection platform, (ii) the scoring method, and (iii) at least two positivity thresholds, so that estimates generated with different cut-offs can be related to one another. The most widely used clone in the sarcoma literature to date is B6H12, used in the two largest series (1242 and 81 specimens), and adopting it prospectively would maximize comparability with existing data. Reporting the continuous distribution of CD47-positive tumor cell percentages, rather than a binary positive/negative call, is essential given the bimodality documented here: a binary classification discards precisely the information that distinguishes the two modes.

A minimum reporting set would therefore comprise: the antibody clone and vendor; the detection platform and signal-amplification method; the tissue type (whole section versus tissue microarray), since core sampling systematically under-represents heterogeneous staining; the numerator and denominator for any proportion reported; and the distribution, not merely the summary, of the percentage of positive tumor cells. Applied retrospectively to the three osteosarcoma cohorts discussed above, such a set would have made it possible to determine whether the six-fold difference in reported positivity reflects biology, tissue sampling, or scoring. Absent it, the difference remains uninterpretable, and any threshold chosen for a future trial is arbitrary rather than evidence-based.

For trial design. Patient selection should be biomarker-guided rather than histology-agnostic. The subtypes with consistently high expression in the available data—chordoma, angiosarcoma, pleomorphic and dedifferentiated liposarcoma, and epithelioid sarcoma—are individually rare, which argues for a basket design with pre-specified subtype strata and mandatory baseline CD47 assessment, rather than a single pooled soft-tissue sarcoma cohort. Where combination is planned, the partner should be selected on a documented mechanistic rationale rather than availability—for example, an agent that induces calreticulin exposure and thereby supplies an “eat me” signal to complement CD47 blockade [14], and the trial should include an internal comparator that permits the incremental contribution of CD47 blockade to be estimated; the experience of NCT04996004, where the reported response fell within the published range for doxorubicin monotherapy, illustrates the cost of omitting this. Dose sequencing is a further design variable that has received little attention: the temporal relationship between chemotherapy administration, which induces calreticulin exposure, and CD47 blockade may critically influence efficacy, and physiologically based pharmacokinetic models integrating tumor CD47 density, erythrocyte sink capacity and FcγR-mediated clearance could inform rational scheduling.

For preclinical reporting. Future sarcoma studies should report randomization, blinding, sample-size justification, and pre-specified dose and endpoints. This review found these items reported in 42%, 32%, 13% and 24% of studies respectively—a level of reporting that makes risk of bias impossible to assess rather than merely uncertain. At minimum, the host immune status of the model should be stated explicitly, because it determines which mechanistic questions the experiment can address.

### 4.4. Comparison with Previous Work

Earlier reviews of CD47-directed therapy in solid tumors have largely treated sarcoma as one entity within a broader oncological landscape, and have emphasized the consistency of preclinical efficacy. The present review reaches a different emphasis for two reasons that are methodological rather than interpretive. It treats expression, preclinical and clinical evidence as separate domains with separately graded certainty (Table 3), which avoids the inferential transfer that occurs when these designs are summarized together. It also maps evidence to sarcoma subtype (Figure 3), making visible a distribution that aggregate reviews obscure: 35 of 50 studies (70%) concern osteosarcoma, and ten of twelve subtypes have no interventional evidence of any kind.

The osteosarcoma predominance deserves emphasis because it has a specific inferential consequence rather than a merely descriptive one. Osteosarcoma is the subtype in which CD47 expression is most contested (87.7% versus 14.3% in different cohorts) and in which the prognostic direction is reported as adverse. It is therefore the subtype for which the evidence base is simultaneously largest and least internally consistent. Conversely, the subtypes with the most consistent high expression—chordoma and angiosarcoma—contribute essentially no preclinical efficacy data, because they are rare and lack established cell lines and models. The practical implication is that the subtypes best supported by expression data are the least supported by functional data, and vice versa; any translation strategy must confront this mismatch directly rather than assume it away.

The prognostic literature is the one area where this review identifies genuine contradiction in the primary data, rather than absence of data. In a meta-analysis across solid tumors, high CD47 expression was consistently associated with poorer survival [42]; in sarcoma, however, the direction is not uniform. In osteosarcoma, CD47 expression was associated with worse outcome; in soft-tissue leiomyosarcoma, high expression was associated with better survival (HR 0.76, 95% CI 0.08–0.88); and in myeloid sarcoma it was reported as a favorable prognostic factor. These findings derive from small, single-center cohorts—the leiomyosarcoma estimate rests on 55 patients, and its confidence interval is wide—so the contradiction between subtypes is present in the reported data but should not yet be interpreted as established biology. It does, however, mean that a unidirectional prognostic narrative for CD47 in sarcoma is not currently defensible, and that the assumption of a straightforward “higher CD47, worse outcome” relationship—which underpins much of the therapeutic rationale—remains unverified in this disease family.

### 4.5. Broader Context: What the CD47 Class Has Taught So Far

Sarcoma is not the first disease in which CD47-directed therapy has been tested, and the wider experience is directly informative for interpreting the sarcoma data. The discontinuation of magrolimab should not be read as a verdict on the antibody class as a whole: lemzoparlimab, an epitope-engineered anti-CD47 antibody with a differentiated erythrocyte-binding profile, shows that the limitations attributed to magrolimab need not be generalized to every agent in this class. Both observations below are drawn from the non-sarcoma literature and should be read as context rather than as findings of this review; no sarcoma-specific evidence for them was identified in this systematic search. The first is that monotherapy activity has been limited, with responses reported mainly in hematological malignancies—for example in higher-risk myelodysplastic syndromes, where magrolimab combined with azacitidine produced encouraging response rates in an early-phase study [43] before the phase 3 program was discontinued [13]—and in selected solid tumors; the second is that the most convincing signals have come from combinations, particularly with a tumor-opsonizing antibody, where CD47 blockade removes the inhibitory signal while the second agent supplies the pro-phagocytic one. Both observations are consistent with the preclinical pattern documented here: in sarcoma, every combination tested outperformed single-agent CD47 blockade, and no study reported that adding blockade was detrimental.

This parallelism cuts both ways. On one hand, it suggests that the sarcoma preclinical literature is mechanistically coherent with the wider field rather than idiosyncratic, which supports the plausibility of the target. On the other, it implies that the sarcoma field should not expect a different outcome from the same trial architecture—a biomarker-unselected monotherapy study—than other solid tumors have obtained. The rationale for testing CD47 blockade in sarcoma rests on the expression data, which are subtype-restricted and bimodal, rather than on the general assumption that the target is broadly overexpressed. This is the reasoning behind the biomarker-led design proposed in Section 4.3.

It is also worth noting what this review did not find. Despite a substantial preclinical literature on resistance mechanisms—including antigen escape, macrophage reprogramming and compensatory innate checkpoints—almost none of these hypotheses has been tested in sarcoma specifically, and none has been tested clinically. The resistance literature in sarcoma is currently extrapolated from other tumor types rather than generated within it.

### 4.6. Strengths and Limitations

Strengths. The search covered three bibliographic databases and three trial registers without language restriction, and all 182 reports that passed title/abstract screening were retrieved in full text and assessed. A record-level adjudication file retains the supporting evidence for every eligibility decision, and the trial-register searches converged with the bibliographic searches (all five sarcoma-indication records identified through WHO ICTRP were already retrieved through ClinicalTrials.gov), providing an internal consistency check on the search. Synthesis followed SWiM, and no pooled estimate is reported.

Limitations. The evidence base is concentrated—35 of the 50 studies concern osteosarcoma—so subtype-level conclusions rest on thin data rather than being generalized, and only two interventional trials met the eligibility criteria, so the clinical conclusions describe an early-phase field that will need revision as trials report. Four included records are conference abstracts and none are preprints; abstract-derived data are less complete, and where an abstract and a subsequent full publication described the same study only the full report was retained. Risk-of-bias appraisal relied on published reporting rather than on investigator contact, so items recorded as “not reported” may reflect incomplete reporting rather than absent safeguards. The searches closed on 14 September 2026: because more than half of the studies appeared in 2023 or later, the field is actively developing and these conclusions should be expected to require updating rather than to remain definitive. Independence is also constrained. Two expression series were recruited at the same department over overlapping periods and may share patients, five preclinical reports come from a single imaging program, and three further reports from one group share a common Ewing sarcoma model; the count in Table S8 should therefore be read as an upper bound on independent evidence. No included study reported a formal interaction analysis, so combination effects are described as additive rather than synergistic.

### 4.7. Implications and Outlook

The position of this field can be stated in three parts: CD47 is a validated innate immune checkpoint with a well-characterized mechanism; it is expressed in a definable subset of sarcomas; and it has not yet been tested in sarcoma in a design capable of detecting a treatment effect. The gap is therefore not in the biology but in the trial architecture and the assay, both of which are addressable within the existing evidence base rather than requiring new discoveries. Nine of ten strategies targeting this axis have no sarcoma-specific clinical data; the immediate priority is to establish a harmonized assay, define the subtypes in which expression is reliably high, and test a biomarker-selected population with an internal comparator, rather than to add further preclinical permutations.

## 5. Conclusions

CD47–SIRPα is a mechanistically well-characterized innate immune checkpoint, and the preclinical literature in sarcoma is both substantial (38 studies) and internally consistent: blockade increased macrophage-mediated phagocytosis, and combination with a partner modality consistently outperformed single-agent blockade. The clinical picture is entirely different. Only two interventional trials have enrolled sarcoma patients, both were single-arm, and one was terminated without posted results; across ten pharmacological strategies identified in this review, nine have no sarcoma-specific clinical data of any kind.

The evidence does not support a conclusion for or against the clinical use of CD47-directed therapy in sarcoma, and we do not draw one. It does support three more specific conclusions. First, CD47 expression is subtype-dependent and bimodal, so the assumption of uniform overexpression across sarcoma—which underpins most of the therapeutic rationale—is not supported by the available data. Second, reported positivity differs more than six-fold between cohorts of the same subtype, which makes cross-study comparison uninterpretable until assay and scoring are harmonized; establishing that standard is a prerequisite for using CD47 as either a prognostic or a predictive biomarker. Third, the preclinical models used most frequently cannot test the mechanism being invoked, because 45% of studies used human tumor cells in immunodeficient hosts.

The rate-limiting step in this field therefore lies in the design of the studies that test the target rather than in its biology. The highest-value next step is a biomarker-selected trial with pre-specified subtype strata and an internal comparator, preceded by a harmonized assay—not a further expansion of preclinical permutations.

## Figures and Tables

**Figure 1:**
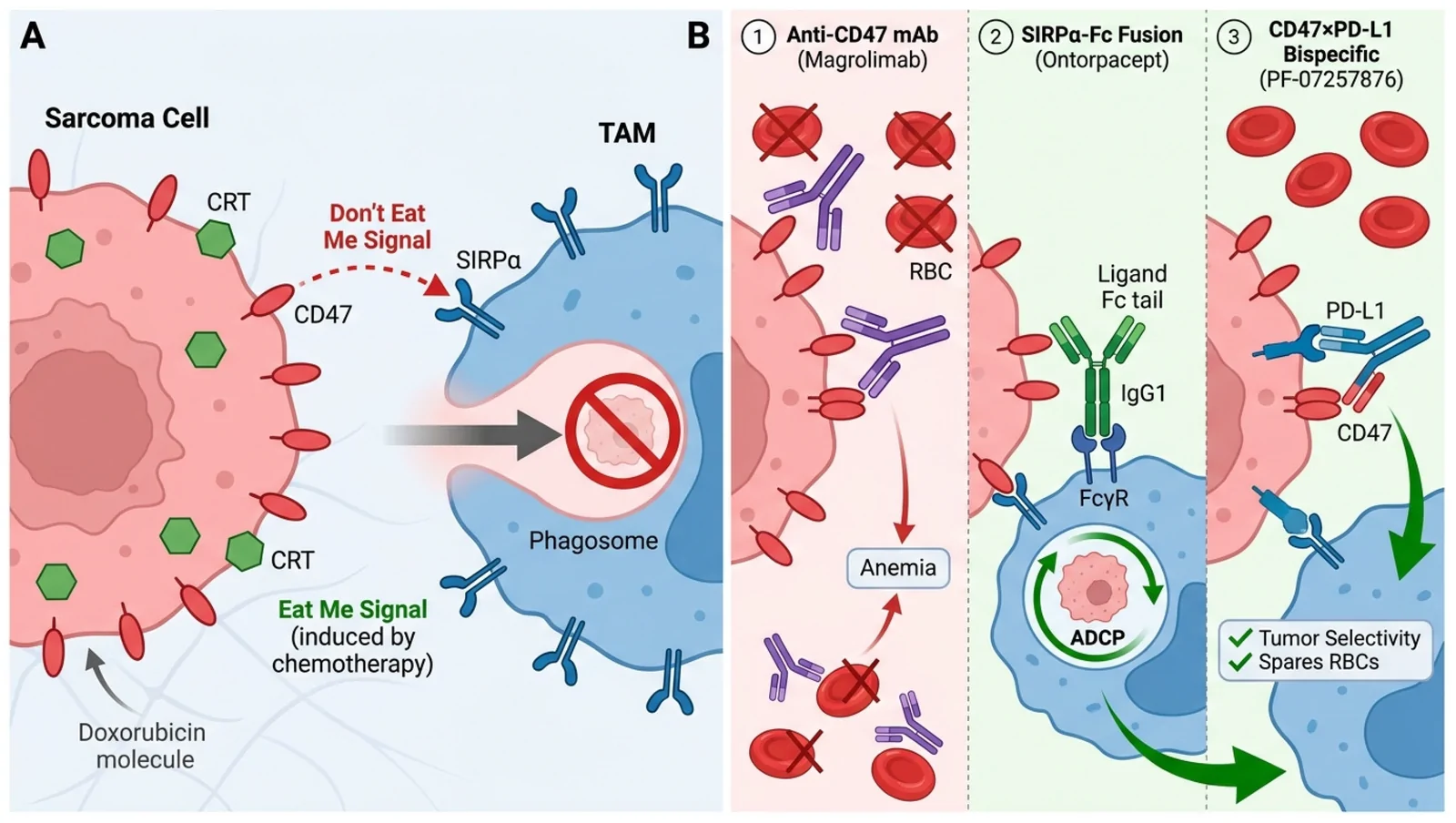
CD47–SIRPα immune evasion mechanism and therapeutic blockade strategies in sarcoma. (**A**) CD47 on sarcoma cells engages SIRPα on tumor-associated macrophages (TAMs), delivering a “don’t eat me” signal; chemotherapy-induced calreticulin (CRT) provides the complementary “eat me” signal. (**B1**) Anti-CD47 monoclonal antibodies (Fc-silent IgG4; magrolimab). (**B2**) SIRPα-Fc fusion proteins (intact IgG1 Fc). (**B3**) CD47 × PD-L1 bispecific antibodies.

**Figure 2:**
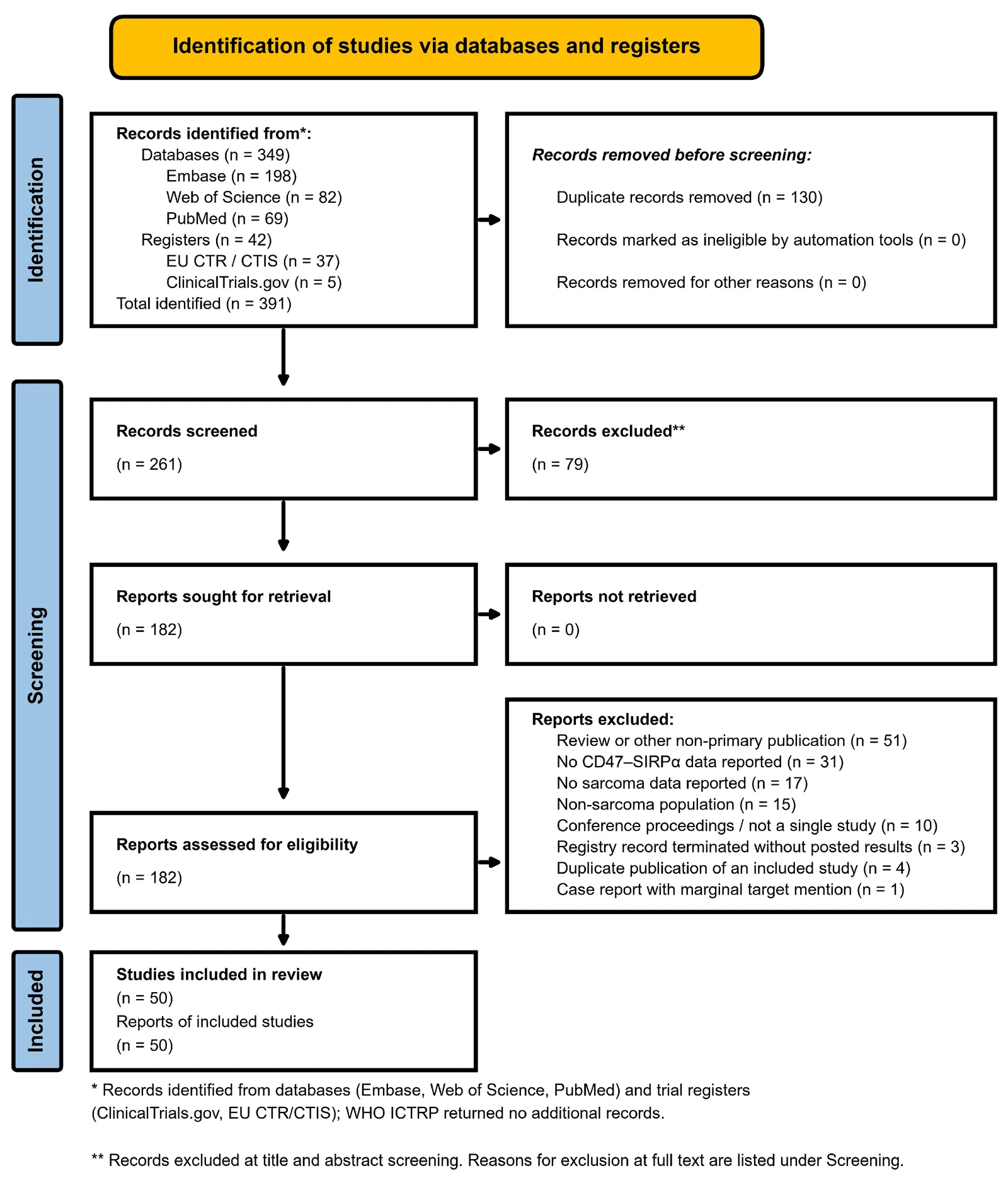
PRISMA 2020 flow diagram of study selection. Records were identified from three bibliographic databases and three trial registers (searched 14 September 2026) and screened at title/abstract and full-text level; 50 studies were included as separate analysis units.

**Figure 3:**
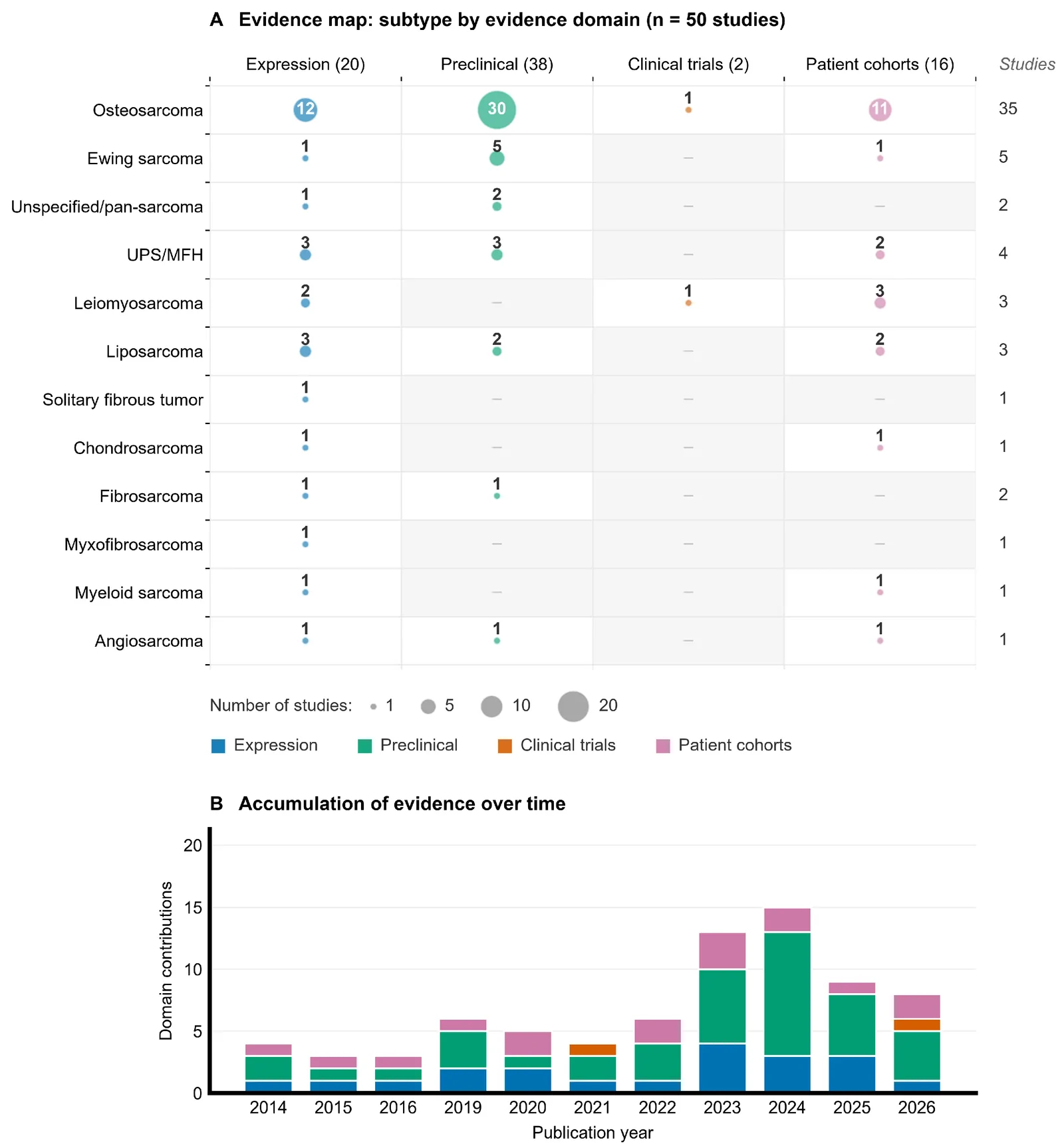
Evidence map of CD47–SIRPα research in sarcoma (*n* = 50 studies). (**A**) Included studies by sarcoma subtype (rows) and evidence domain (columns); bubble areas and numerals give study counts, and “–” marks subtype–domain combinations with no evidence. (**B**) Temporal accumulation of evidence by domain. A single study may contribute to more than one subtype and domain, so totals exceed 50. UPS, undifferentiated pleomorphic sarcoma.

**Figure 4:**
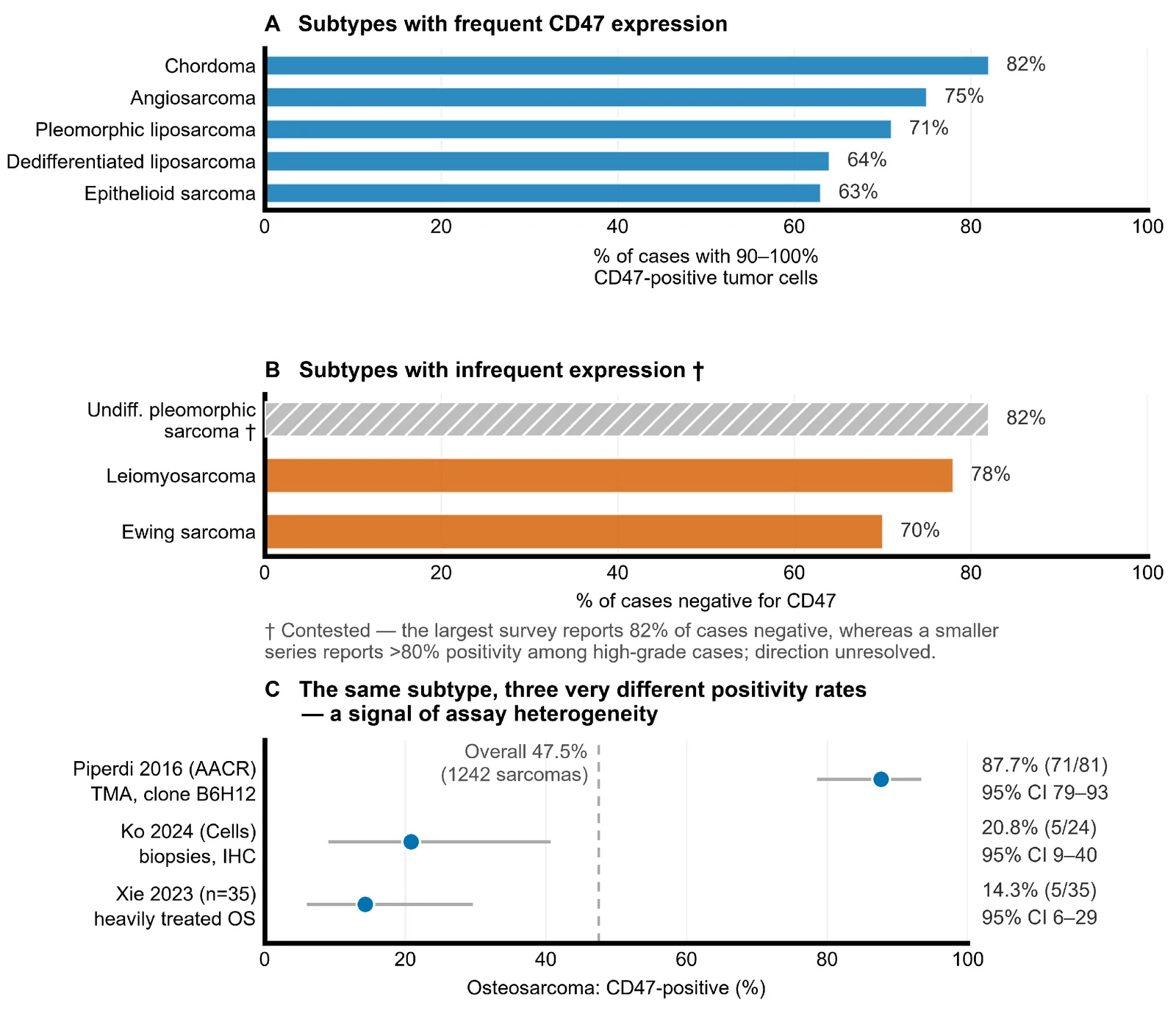
Heterogeneity of CD47 expression across sarcoma subtypes and studies. (**A**) Cases with 90–100% CD47-positive tumor cells among frequently expressing subtypes. (**B**) Cases negative for CD47 among infrequently expressing subtypes; undifferentiated pleomorphic sarcoma (hatched) is contested and should not be read as a low expresser (Section 3.2.1). (**C**) CD47 positivity in osteosarcoma in three independent cohorts (whiskers, Wilson 95% CI) [21,22,23]. Panels (**A**,**B**) derive from a single survey of 1242 specimens and use different metrics, so they should not be compared directly. No pooled estimate is presented.

**Figure 5:**
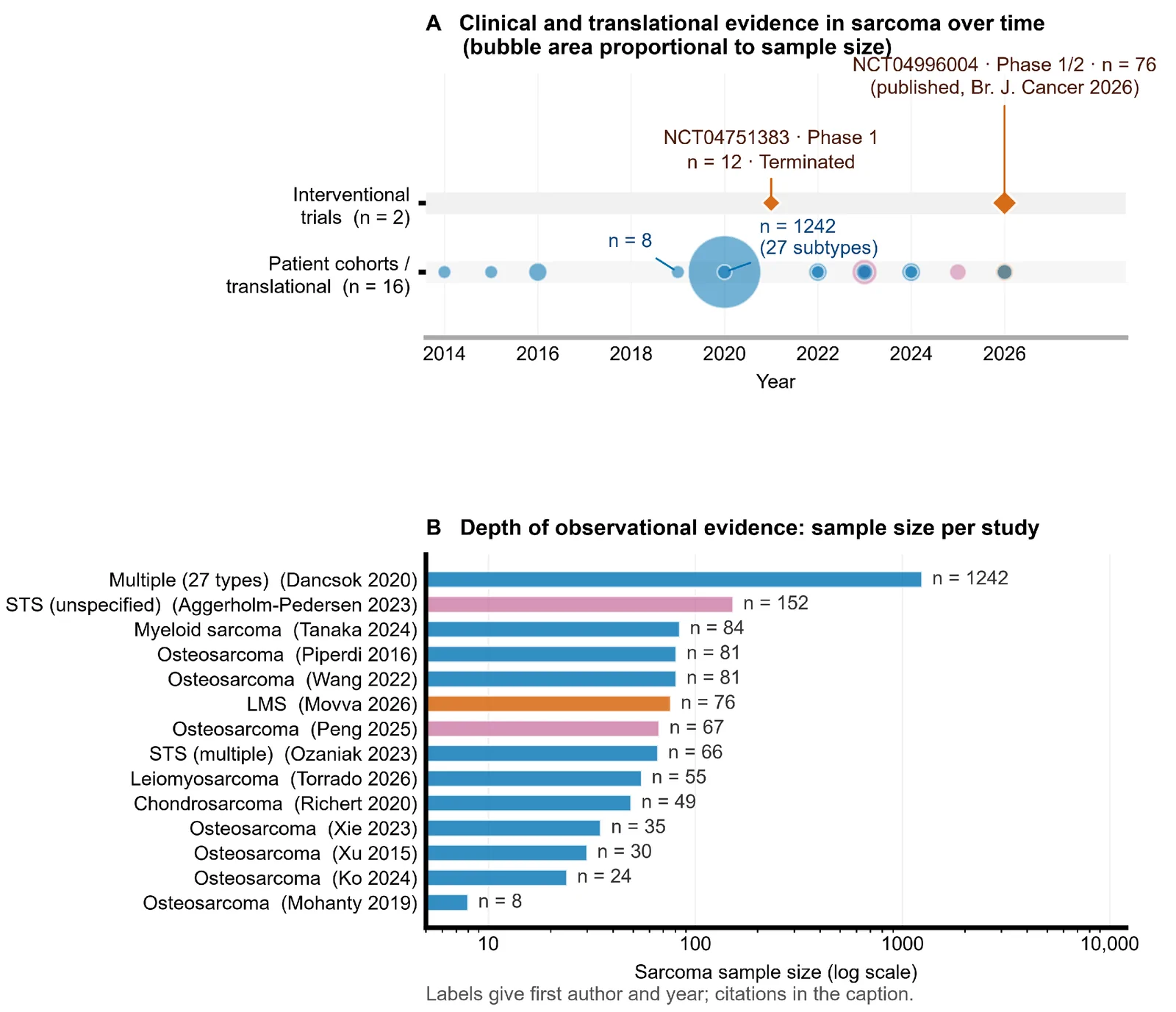
Clinical and translational evidence for CD47–SIRPα targeting in sarcoma. (**A**) Temporal distribution of the two interventional trials (upper row) and the 16 patient-cohort/translational records (lower row); trials are annotated with registry number, phase, sarcoma enrollment and availability of posted results. (**B**) Sarcoma sample size per patient-cohort study (logarithmic scale), color-coded by specimen source. Numbers in square brackets are reference numbers, not sample sizes. No pooled estimate is presented [15,20,21,22,23,25,26,27,28,29,34,35,36,37].

**Table 1:** CD47 expression by sarcoma subtype, as reported in the source studies.

Subtype	CD47 Expression	Reported Finding	Ref.
Chordoma	High	90–100% CD47^+^ cells in 82% of cases	[20]
Angiosarcoma	High	90–100% CD47^+^ cells in 75% of cases	[20]
Pleomorphic liposarcoma	High	>90% CD47^+^ cells in 71% of cases	[20]
Dedifferentiated liposarcoma	High	>90% CD47^+^ cells in 64% of cases	[20]
Epithelioid sarcoma	High	>90% CD47^+^ cells in 63% of cases	[20]
Leiomyosarcoma	Low	78% of cases CD47-negative	[20]
Ewing sarcoma	Low	70% of cases CD47-negative	[20]
Undifferentiated pleomorphic sarcoma	Contested	82% negative [20] vs. >80% moderate-to-diffuse positive [24]; strongest ex vivo anti-CD47 response in UPS [25]	[20,24,25]
Osteosarcoma	Conflicting	Positivity 87.7% [21], 20.8% [22] and 14.3% [23] across three cohorts	[21,22,23]

No pooled estimate is presented. High- and low-expression calls derive from a single immunohistochemical survey of 1242 specimens (clone B6H12) [20]; undifferentiated pleomorphic sarcoma is recorded as contested (Section 3.2.1) and osteosarcoma as conflicting (Section 3.2.2), because the source studies disagree.

**Table 2:** Pharmacological strategies targeting the CD47–SIRPα axis in sarcoma.

Strategy	Preclinical Studies (n)	Sarcoma Clinical Evidence	Rationale/Key Finding
CD47–SIRPα blockade as monotherapy (or biology characterization only)	14	No interventional evidence	Activity observed in osteosarcoma, Ewing sarcoma and UPS models; no sarcoma-specific monotherapy trial has reported results
Nanocarrier	11	No sarcoma-specific trial identified	Improved tumor selectivity and reduced systemic exposure; combining CD47 blockade with a pro-apoptotic or immunogenic-cell-death stimulus
Radiotherapy/ablation	5	No sarcoma-specific trial identified	CD47 upregulation after irradiation; blockade restores CD8^+^ T-cell cooperation
Calreticulin/ICD induction	8	No sarcoma-specific trial identified	Additive pro-phagocytic “eat-me” signaling on top of CD47 blockade
Chemotherapy	4	Yes—1 trial (NCT04996004, ontorpacept + doxorubicin, LMS)	Chemotherapy-induced CD47 upregulation provides a mechanistic rationale for sequencing or combination
Second immune checkpoint	3	No—anti-GD2/CD47 and anti-CD99 data are preclinical only	Innate (CD47) plus adaptive (PD-1/PD-L1) or tumor-antigen (GD2, CD99) co-targeting
CAR-T/engineered cellular therapy	2	No sarcoma-specific trial identified	CD47 blockade can deplete engineered T cells via on-target off-tumor binding—a therapeutic conflict that requires engineered CD47
Targeted inhibitor (STAT3/kinase)	2	No sarcoma-specific trial identified	Pathway-level CD47 regulation (e.g., STAT3, LAT2) offers an indirect route to lowering CD47
Oncolytic virus	2	No sarcoma-specific trial identified	Virus-induced innate activation combined with removal of the “don’t eat me” signal
Innate immune agonist (LPS/TLR)	1	No sarcoma-specific trial identified	Direct macrophage priming combined with CD47 blockade

Strategies are ordered by preclinical study count and overlap, so counts exceed the 38 preclinical studies included. “No sarcoma-specific trial identified” means that no sarcoma-enrolling trial tested that combination. No pooled estimate is presented.

**Table 3:** Certainty of evidence by domain.

Evidence Domain	Risk of Bias	Indirectness	Certainty
Expression profiling	Moderate	Low	Low
Patient cohorts	Moderate–High	Low	Low
Interventional trials	High	High	Very Low
Preclinical	High	High (24% patient-derived/humanized)	Very Low

Ratings were assigned with a GRADE-informed framework; they follow from the measured reporting frequencies in Tables S2 and S4 and from the domain-level assessments in Table S5.

## Data Availability

All data extracted and analyzed in this review are available within the article and its Supplementary Materials. The search strategies, the risk-of-bias and certainty appraisals, and the per-study characteristics of all included studies are provided in the Supplementary Materials (Tables S1–S10). The tabulated data underlying all numbered Supplementary Tables (Tables S2–S10) are additionally provided. No analytic code was used (narrative synthesis per SWiM).

## Supplementary Materials

The following supporting information can be downloaded at: https://www.mdpi.com/article/10.3390/biomedicines14102187/s1, Table S1. Complete search strategies for all databases, verbatim as executed. Table S2. Risk-of-bias appraisal—expression-profiling and patient-cohort studies. Table S3. Risk-of-bias appraisal—interventional trials. Table S4. Model fidelity and reporting quality—preclinical studies. Table S5. Basis for the certainty ratings reported in Table 3. Table S6. Reasons for exclusion at full-text assessment. Table S7. Characteristics of the expression-profiling and patient-cohort studies. Table S8. Characteristics of the included preclinical studies. Table S9. Interventional trials enrolling sarcoma patients. Table S10. Patient-cohort and translational studies using human sarcoma material. Table S11: PRISMA checklist. References [35,36,37,44,45,46,47,48,49,50,51,52,53,54,55,56,57,58,59,60,61,62,63,64,65,66,67,68,69,70,71,72,73,74] are cited in the Supplementary Materials.

## Abbreviations

The following abbreviations are used in this manuscript:

ADCP	Antibody-Dependent Cellular Phagocytosis
CRT	Calreticulin
DDLPS	Dedifferentiated Liposarcoma
ICD	Immunogenic Cell Death
LMS	Leiomyosarcoma
PBPK	Physiologically Based Pharmacokinetic
SIRPα	Signal Regulatory Protein alpha
STS	Soft-Tissue Sarcoma
TAM	Tumor-Associated Macrophage
UPS	Undifferentiated Pleomorphic Sarcoma
WDLPS	Well-Differentiated Liposarcoma
