# Supplementary material for: CD47–SIRPα as a Therapeutic Target in Sarcoma: A Systematic Review of Expression, Preclinical, and Clinical Evidence
Source: Biomedicines. 2026 Sep 27;14(10):2187. doi: 10.3390/biomedicines14102187 (PMC13701423; doi:10.3390/biomedicines14102187)
Supplement: Supplementary file 1 [file biomedicines-14-02187-s001.zip › Tables 1-10.pdf]

Supplementary Materials

Table S1. Complete search strategies for all databases, verbatim as executed. Summary by source.

#	Source	Strategy Structure	Records	Search Date
1	Embase	#36 (three axes combined with AND; full chain below)	198	14 Sep 2026
2	Web of Science Core Collection	TS= target axis AND sarcoma axis (verbatim string below)	82	14 Sep 2026
3	PubMed	S1 AND S2 AND date (below)	69	13 Sep 2026; re-checked 14 Sep 2026
4	ClinicalTrials.gov	query.cond × query.intr (below)	5	13 Sep 2026; re-checked 14 Sep 2026
5	EU CTR (legacy)	30 target terms, each applied separately as OR	20	14 Sep 2026
6	EU CTIS	containAny blocks A and B (below)	17	14 Sep 2026
7	WHO ICTRP	intervention-field OR string	0	13 Sep 2026; re-checked 14 Sep 2026
	**Total**		**391**	

Database subtotal **349** (Embase 198 + Web of Science 82 + PubMed 69); trial-register subtotal **42** (EU CTR 20 + EU CTIS 17 + ClinicalTrials.gov 5).

Web of Science Core Collection — verbatim strategy

Executed in **Advanced Search**; the string below was pasted as a single expression.

TS=(("CD47" OR "SIRPalpha" OR "SIRP-alpha" OR "SIRPA" OR "SIRPG" OR "integrin-associated protein" OR "integrin associated protein" OR "signal regulatory protein" OR "CD47-SIRPalpha" OR "CD47/SIRPalpha" OR "phagocytosis checkpoint" OR "don't eat me" OR "do not eat me" OR "magrolimab" OR "Hu5F9-G4" OR "ontorpacept" OR "TTI-621" OR "TTI-622" OR "evorpacept" OR "ALX148" OR "lemzoparlimab" OR "IBI188" OR "HCB101" OR "HCB301" OR "PF-07257876" OR "IMM2520" OR "maplirpacept" OR "TQB2928" OR "AO-176" OR "CC-95251" OR "ZL-1201" OR "AK117" OR "SHR-1802" OR "BI 765063" OR "OSE-172" OR "BAY-1834942" OR "SL-172154" OR "DSP107")
AND
("sarcoma" OR "sarcomas" OR "soft tissue sarcoma" OR "soft-tissue sarcoma" OR "osteosarcoma" OR "bone sarcoma" OR "leiomyosarcoma" OR "liposarcoma" OR "DDLPS" OR "WDLPS" OR "rhabdomyosarcoma" OR "Ewing sarcoma" OR "Ewing's sarcoma" OR "synovial sarcoma" OR "undifferentiated pleomorphic sarcoma" OR "UPS" OR "myxofibrosarcoma" OR "angiosarcoma" OR "chondrosarcoma" OR "myeloid sarcoma" OR "malignant fibrous histiocytoma" OR "MPNST" OR "malignant peripheral nerve sheath tumor" OR "epithelioid sarcoma" OR "fibrosarcoma" OR "pleomorphic liposarcoma" OR "myxoid liposarcoma"))

Date range: Custom date range, 2014-01-01 to the search date (14 Sep 2026).

Editions searched (all selected): Science Citation Index Expanded (SCI-EXPANDED); Social Sciences Citation Index (SSCI); Emerging Sources Citation Index (ESCI); **Conference Proceedings Citation Index – Science (CPCI-S)**; Conference Proceedings Citation Index – Social Science & Humanities (CPCI-SSH). CPCI-S was selected deliberately: ASCO and CTOS conference abstracts are indexed there, and these abstracts are an eligible source of study reports in this review.

Records identified: **82**.

Embase — complete search chain (#36 , N = 198)

Final line (#36):

(#4 OR #17 OR #21 OR #22) AND (#35 OR #6 OR #7)

AND [embase]/lim AND [01-01-2014 to 30-09-2026]/pd

Target axis

- #1 — 'cd47' → 8,846
- #2 — 9 CD47/SIRPα synonyms as :ti,ab,kw (applied term by term) → 7,957
- #3 — 28 drug names as :ti,ab,kw (applied term by term) → 763
- **#4** — #1 OR #2 OR #3 → **10,454**
- #17 — 5 concept terms + 5 drug names as :de (term by term) → 1,741
- #18 — #4 OR #17 → 10,869
- #20 — 'signal regulatory protein':de → 793
- #21 — remaining 6 concept terms as :de → 123
- #22 — remaining 23 drug names as :de → 133

Disease axis — the only change from the registered strategy

- #5 — 'sarcoma'/exp → 329,995 — **abandoned** (retrieves a DLBCL subtree)
- #6 — 14 terms as :ti,ab,kw (term by term) → 224,333 — retained
- #7 — 12 terms as :ti,ab,kw (term by term) → 31,561 — retained
- #8 — #5 OR #6 OR #7 → 385,636
- #32 — 'sarcoma'/exp AND 'diffuse large b cell lymphoma'/exp → **47,019** — magnitude of the overlap
- **#35** — 'sarcoma'/exp NOT 'diffuse large b cell lymphoma'/exp → **282,976 — adopted**

(329,995 − 47,019 = 282,976; difference 0.)

Final set and limits

- #9 — #4 AND #8 → 429
- #10 — #9 AND [embase]/lim → 396
- #11 — #10 AND [2014-2026]/py → 380
- #13 — #10 AND [01-01-2014 to 30-09-2026]/pd → 372 (protocol version)
- #36 — final line as above → **198 = N**
- #43 — #4 AND (#35 OR #6 OR #7) AND [embase]/lim AND /pd → 182
- #41 — #36 AND [conference abstract]/lim → **36** (conference abstracts)
- #42 — #36 NOT [medline]/lim → **98** (Embase-unique)

Arithmetic closure: |#4 ∪ #8| = 429 (difference 0); 429 − 396 = 33 (database limit); 396 − 372 = 24 (date granularity); #36 − #43 = 16.

PubMed

S1 AND S2 AND ("2014/01/01"[Date - Publication] : "2026/09/14"[Date - Publication])

- **S1 (target axis)** = concept terms OR drug names, using both [Title/Abstract] and [MeSH Terms]
- **S2 (sarcoma axis)** = 24 subtype terms, using both [Title/Abstract] and [MeSH Terms]
- Observed: S1 alone 4,555; S2 alone 288,894; S1 AND S2 without the date limit 75; **with the date window 69**.

Trial registers

ClinicalTrials.gov

- query.cond = 16 sarcoma indication terms (OR)
- query.intr = 20 CD47/SIRPα axis terms and drug names (OR)
- Retrieved: **5** (NCT04751383, NCT04996004, NCT06438783, NCT04861948, NCT02890368)

EU CTR (legacy)

- 30 target terms applied **individually** with OR: CD47, SIRPalpha, SIRPα, SIRPA, magrolimab, Hu5F9-G4, ontorpacept, TTI-621/622, evorpacept, ALX148, lemzoparlimab, IBI188, HCB101/301, PF-07257876, IMM2520, maplirpacept, TQB2928, AO-176, CC-95251, ZL-1201, AK117, SHR-1802, BI 765063, OSE-172, BAY-1834942, DSP107, IMC-002, ES-004, SL-172154
- Retrieved **20**; none sarcoma-related

EU CTIS (public API)

- Block A (CD47 axis): containAny accepts at most 23 terms per request, so it was run in 2 batches; union = **17**
- Block B (sarcoma indications): medicalCondition, terms applied individually with OR
- Intersection = **0**

WHO ICTRP

- Advanced Search, intervention-field OR string: **212 records / 153 trials**, of which **5** were sarcoma indications
- All 5 were identical, record for record, to those already retrieved from ClinicalTrials.gov, so the net addition was 0 (see S1.6), and ICTRP is therefore recorded as contributing no additional records in Figure 2

Three departures from the PROSPERO v3.0 registered wording (disclosed per PRISMA-S)

1. **Registered:** Field label attached to a **parenthetical group** ((A OR B):de) **Executed:** Changed to **term-by-term attachment** (A:de OR B:de) **Reason:** Under Embase/Ovid syntax, group attachment changes the semantics of the search
2. **Registered:** Granularity of the **year limit** unspecified **Executed:** Standardized to **day granularity**, [01-01-2014 to 30-09-2026]/pd **Reason:** To align the three databases across four dimensions: date granularity, language, publication type, and database scope
3. **Registered:** Disease axis 'sarcoma'/exp **Executed:** Changed to **'sarcoma'/exp NOT 'diffuse large b cell lymphoma'/exp** **Reason:** This Emtree explosion retrieves a DLBCL subtree (measured overlap **47,019 records**); the proportion of records without sarcoma evidence fell from **50.4% to 2.5%**

Note on WHO ICTRP. The basic ICTRP search box does not support Boolean operators (CD47 AND sarcoma is parsed as an AND of three separate words and necessarily returns 0). Using **Advanced Search** retrieved 212 records / 153 trials, of which 5 were sarcoma indications; all 5 matched records already retrieved from ClinicalTrials.gov. The net addition was therefore 0, and ICTRP is not shown separately in the PRISMA flow diagram.

Availability of verbatim search strings

Full verbatim search strings for every source, together with the session-level search histories, are available from the corresponding author on reasonable request.

Table S2. Risk-of-bias appraisal—expression-profiling and patient-cohort studies. Item-level reporting frequency for the six modified Newcastle–Ottawa items across the 20 included expression-profiling and patient-cohort studies (median 4 of 6 items met, range 0–6). Per-study appraisals, each accompanied by the source sentence on which it rests, are provided in the table below.

NOS Item	Reported	Interpretation
Cohort representativeness	13/20 (65%)	Predominantly single-center and retrospective
Sample size	19/20 (95%)	—
Assay described	9/20 (45%)	Clone and detection platform frequently unspecified — the weakest item alongside confounding
Positivity threshold defined	17/20 (85%)	Better reported than the assay itself
Control for confounders	9/20 (45%)	Unadjusted confounding — the weakest item
Outcome statistics	14/20 (70%)	—

Table S3. Risk-of-bias appraisal—interventional trials. Appraisal of the two interventional trials that enrolled sarcoma patients (n = 2). Both were single-arm and non-randomized; the independent contribution of CD47–SIRPα blockade to the reported response therefore cannot be isolated. Presence of a control arm and availability of posted results were assessed from the registry entry and the full report.

**Trial**	**Design**	**Randomization**	**Control Arm**	**Results Posted**
NCT04751383 (magrolimab + dinutuximab)	Phase 1 dose-finding	No (registry entry states NON_RANDOMIZED)	None	No (terminated)
NCT04996004 (ontorpacept + doxorubicin)	Phase 1/2, single-arm, two-stage	No	None	Yes

Table S4. Model fidelity and reporting quality—preclinical studies. Reporting quality and model fidelity of the 38 included preclinical studies. **(A)** SYRCLE-informed checklist items, scored as reported or not reported. **(B)** Model fidelity, classified as high (patient-derived or humanized), moderate (human cell line in an immunodeficient host, or murine model in an immunocompetent host) or low (in vivo model not specified, or in vitro only). Tumor origin and host immune status are recorded as two orthogonal dimensions rather than collapsed into a single score, because they constrain translational inference in different ways. Per-study data are provided in the table below.

(A)

**Item**	**Reported**
In vivo validation	37/38 (97%)
Randomization	16/38 (42%)
Blinding	12/38 (32%)
Sample-size justification	5/38 (13%)
Pre-specification of dose and endpoint	9/38 (24%)

(B)

Fidelity	Studies
High (patient-derived / humanized)	9
Moderate (human cells in immunodeficient host)	17
Moderate (murine, immunocompetent)	4
Low (unspecified in vivo)	7
Low (cell line / in vitro)	1

Only **9 of 38 studies (24%)** used patient-derived or humanized models; **14 of 38 (37%)** implanted human sarcoma cell lines into immunodeficient hosts — models that carry human tumor cells but not a human immune system; a further **8 of 38 (21%)** were of low fidelity (in vivo model unspecified, or in vitro only). Because CD47–SIRPα blockade is proposed to act through macrophage–T-cell crosstalk, reliance on immunodeficient xenografts is a substantive constraint on the translational claims that can be drawn from this domain, rather than a technicality.

Table S5. Basis for the certainty ratings reported in Table 3. Domain-level basis for the certainty ratings in Table 3, giving the risk of bias, indirectness, inconsistency and imprecision assessed for each evidence domain. Imprecision is graded on the GRADE scale (**no serious**/**serious**/**very serious**), against a starting certainty of *Low* for observational evidence. Certainty is reported per domain and is not aggregated: the ratings follow directly from the reporting frequencies set out in Tables S2–S4 and from the model composition of the underlying literature, rather than from editorial conservatism.

Evidence Domain	Risk of Bias	Indirectness	Inconsistency	Imprecision	Certainty
Expression profiling	Moderate (clone and threshold not standardized)	Low	Low	No serious imprecision	Low

Patient cohorts	Moderate–High (retrospective; confounding unadjusted in 57%)	Low	Moderate	No serious imprecision	Low
Interventional trials	High (non-randomized, no control arm)	High (phase 1/2 only)	—	Very serious imprecision (one trial without results)	Very Low
Preclinical	High (randomization 42%, blinding 32%)	High (only 24% patient-derived or humanized)	Moderate	Serious imprecision (sample-size justification in 13%)	Very Low

Table S6. Reasons for exclusion at full-text assessment. Reasons for exclusion of the 132 reports assessed in full text. Categories are mutually exclusive; each excluded record was assigned to a single category. A record-level audit trail is retained in the screening database.

Reason for Exclusion	Reports (n)
Review or other non-primary publication	51
No CD47–SIRPα data reported	31
No sarcoma data reported	17
Non-sarcoma population	15
Conference proceedings / not a single study	10
Registry record terminated without posted results	3
Duplicate publication of an included study	4

Case report with marginal target mention	1
Total excluded at full text	132

These tables give the per-study characteristics underlying the synthesis in Sections 3.2–3.4 of the manuscript.

Table S7. Characteristics of the expression-profiling and patient-cohort studies. Characteristics of the included expression and patient-cohort studies (n = 20). The final column marks studies from which no CD47 result could be extracted with an identifiable source sentence. IHC, immunohistochemistry; HR, hazard ratio. Reference numbers follow the numbering of the main text; studies are ordered by sarcoma subtype and, within subtype, by cohort rather than by reference number.

Ref	Study ID	Subtype	Sample Source	N	Assay Clone	Positivity	Threshold Scoring	Clinical Association	Data Status
[41]	12	not reported	not reported	not reported	not reported	not reported	not reported	not reported	no extractable CD47 result
[35]	41	not reported	not reported	not reported	not reported	not reported	not reported	not reported	no extractable CD47 result
[42]	128	not reported	not reported	not reported	not reported	not reported	not reported	not reported	no extractable CD47 result
[26]	130	Leiomyosarcoma	Tissue microarray (ST-LMS 23; U-LMS 32)	55	not stated	see survival association	spatial RNA–protein (GeoMx DSP)	high CD47 → improved survival in ST-LMS (HR 0.76, 95% CI 0.08–0.88, p = 0.021)	extractable

[45]	133	not reported	not reported	not reported	not reported	not reported	not reported	not reported	no extractable CD47 result
[46]	139	not reported	not reported	not reported	not reported	not reported	not reported	not reported	no extractable CD47 result
[47]	141	not reported	not reported	not reported	not reported	not reported	not reported	not reported	no extractable CD47 result
[48]	151	not reported	not reported	not reported	not reported	not reported	not reported	not reported	no extractable CD47 result
[24]	152	Osteosarcoma	Diagnostic biopsies	24	not stated	20.8% (5/24)	staining intensity high/low	metastasis correlation	extractable
[21]	157	Soft-tissue sarcoma (UPS/MFS/LPS)	FFPE tumor (treatment-naive)	55	not reported (IHC)	>80% of high-grade UPS, 70% MFS, >60% LPS moderate-to-diffuse positive	0/1+/2+/3+ by tumor area	survival (Kaplan–Meier) — no association with grade/metastasis/death	extractable
[27]	159	Myeloid sarcoma	Bone marrow biopsies	84	not stated	see prognostic direction	IHC of CD47 and SIRPα	CD47 = favorable prognostic factor (not independent in multivariable analysis)	extractable
[25]	165	Osteosarcoma	Tumor	35	not	14.3% (5/35)	positive rate	checkpoint co-expression	extractable

		(heavily pre-treated)	tissue		stated				
[22]	166	Soft-tissue sarcoma (multiple)	Resected tissue (ex vivo TIIC culture)	66	not reported	CD47 expressed in 86.9% of patients (mixed STS cohort); strongest ex vivo anti-CD47 response in one UPS	flow cytometry / multiplex immunoassay	ex vivo anti-CD47 / anti-PD-1 cytokine response	extractable
[49]	167	not reported	not reported	not reported	not reported	not reported	not reported	not reported	no extractable CD47 result
[29]	168	Osteosarcoma	Matched pre/post-chemo tissue	81	not stated	upregulated post-chemotherapy	CD47 H-score	associated with patient mortality	extractable
[20]	172	Multiple (24–27 types)	Tissue microarray	1,242	B6H12	47.5% at least focal; bimodal (0% or ≥90%)	% positive tumor cells; intensity excluded	adverse in osteosarcoma; SIRPα^{+} macrophages adverse in SS/MFS	extractable
[43]	175	not reported	not reported	not reported	not reported	not reported	not reported	not reported	no extractable CD47 result

[44]	176	not reported	not reported	not reported	not reported	not reported	not reported	not reported	no extractable CD47 result
[28]	177	Osteosarcoma	Freshly isolated tumor tissue	8 + 30	not stated	CD47 up-regulated	mRNA expression	prognostic (cohort of 30)	extractable
[23]	178	Osteosarcoma	Tissue microarray	81	B6H12	87.7%	positivity by intensity vs controls	prevalence; prognostic value	extractable

Table S8. Characteristics of the included preclinical studies. Characteristics of the included preclinical studies (n = 38). Model fidelity was classified as high (patient-derived or humanized), moderate (human cell line in an immunodeficient host, or murine model in an immunocompetent host) or low (in vivo model not specified, or in vitro only). Key functional findings are drawn from the source reports (abstracts or full texts). NR, not reported. Where a study used both an immunodeficient and an immunocompetent host, immune status is recorded as "Both". Reference numbers follow the numbering of the main text; studies are ordered by sarcoma subtype and, within subtype, by cohort rather than by reference number.

Ref	Study ID	Year	Title	Subtype	Model Types	Tumororigin	Immune Status	Fidelity	Combination Partner	Key Functional Finding
[41]	12	2025	Preclinical Development of SGN-CD47M: Protease-Activated Antibody Technology	Unspecified/pan-sarcoma	Xenograft ＋ Syngeneic ＋Cell line	Human cell line	Both	Moderate (human cells in immunodeficient host)	Monotherapy/not applicable	For these reasons, SGN-CD47M may have enhanced antitumor activity and improved tolerability relative to existing therapies that

			Enables Selective T							target the CD47–signal regulatory protein α interaction.
[71]	17	2024	Engineered CD47 protects T cells for enhanced antitumour immunity.	Osteosarcoma	Humanized＋Orthotopic＋Xenograft＋Cell line	Human cell line	Immunodeficient	High (patient-derived / humanized)	CAR-T / engineered cellular therapy	Here we administered anti-CD47 antibodies in combination with adoptively transferred T cells with the goal of enhancing antitumour efficacy but observed abrogated therapeutic benefit due to rapid macrophage-mediated clearance of T cells expressing chimeric antigen receptors (CARs) or engineered T cell receptors.
[50]	60	2022	996 Functional and	Osteosarcoma	Cell line	Human cell line	In vitro only	Low (cell line / in vitro)	Monotherapy / not applicable	Of note, phagocytic monocytes of

			transcriptomic profiling of myeloid cells in pediatric solid tumors to inform						tumor cells blocked with anti-CD47 had downregulated inflammatory signaling and increased anti-inflammatory PPAR signaling, compared to those from the isotype control.
[42]	128	2014	Abstract PR12: Overcoming immune evasion in pediatric hematologic and solid tumor malignancies:	Osteosarcoma／Ewing sarcoma	Orthotopic＋Xenograft	NR	Immunodeficient	Low (unspecified in vivo)	Monotherapy / not applicable	Furthermore we observe potent anti-tumor efficacy and survival benefit in MB, pHGG, ATRT and PNET orthotopic xenografts models establishing the ability of the huCD47 antibody to across the blood brain barrier by the CD47 blocking antibody.
[56]	129	2025	pH-triggered	Osteosarcoma	Orthotopic＋	Human cell line	Immunoc	Moderate (murine,	Nanocarrier／Calreticulin /	MRPH selectively dissociates in the

			Mn-ZIF8 nanoreactor enhances tumor immunotherapy by amplifying the cGAS-STING pathw		Syngeneic ＋Cell line		ompetent	immunocompetent)	ICD induction	acidic tumor microenvironment, releasing Mn2+ and NO; the two act synergistically to amplify cGAS-STING activation, triggering immunogenic cell death, dendritic cell maturation and CD8+ T cell infiltration while inhibiting CD47-mediated immunosuppression.
[51]	131	2026	Dual role of IFNγ in reprogramming the undifferentiated pleomorphic sarcoma cell line JBT19 towa	UPS/MFH	Humanized＋ Xenograft ＋Cell line	Human cell line	Immunocompetent	High (patient-derived / humanized)	Monotherapy / not applicable	It was identified that IFNγ treatment significantly decreased the proliferation of JBT19 cells and increased the surface expression levels of cluster of differen- tiation (CD)44, CD47, CD95 (Fas), major

										histocompatibility complex (MHC)-I and programmed death-ligand 1 (PD-L1).
[57]	134	2025	Enhancing immunogenicity and release of in situ-generated tumor vesicles for autologous vaccines	Osteosarcoma	Orthotopic＋Syngeneic＋Cell line	Human cell line	Immunocompetent	Moderate (murine, immunocompetent)	Nanocarrier	This approach combines Andrographolide, Icariside II, and shRNA targeting UDP-N-acetylglucosamine 2-epimerase/N-acetylmannosamine kinase (GNE), which accumulates in the tumor region, resulting in the regulation of immunosuppressive pathways and the reduction of sialic acid production.
[52]	135	2024	Extracellular Vesicles from Highly Metastatic	Osteosarcoma	Cell line	Patient-derived	Immunocompetent	High (patient-derived / humanized)	Monotherapy / not applicable	EVs from highly metastatic K7M2 osteosarcoma cells associated with M2 macrophages,

			Osteosarcoma Cells Induce Pro-Tumorigenic Macropha							an interaction abrogated by pre-treatment with anti-CD47 antibody; K7M2-EVs polarized fewer macrophages to M1.
[65]	137	2023	The role of hypoxia and radiation in developing a CTCs-like phenotype in murine osteosarcoma cel	Osteosarcoma	Cell line	Human cell line	Immunocompetent	Low (unspecified in vivo)	Radiotherapy / ablation	It is well documented in literature that high protein levels of CD133 are related to high metastatic capability of cancer cells as well as an increased resistance to radiotherapy treatments (Liou, 2019).
[53]	145	2023	SIRPA enhances osteosarcoma metastasis by stabilizing	Osteosarcoma	Xenograft ＋Cell line	Human cell line	Immunodeficient	Moderate (human cells in immunodeficient host)	Monotherapy / not applicable	Combined treatment with the anti-SIRPA antibody and arginase, which blocked the SP1 stabilization circle,

			SP1 and promoting SLC7A3-mediated arginine							impaired tumor metastasis in mice bearing xenografts formed from SIRPA-overexpressing cells.
[32]	146	2026	Engineering a tumor-homing ZIF-8 nanoplatform for safe and synergistic immunotherapy of osteosar	Osteosarcoma	Cell line	Human cell line	NR	Low (unspecified in vivo)	Nanocarrier	In vitro and in vivo, OTP@ZIF-8 synergistically inhibited tumor growth by remodeling the immunosuppressive microenvironment, decreasing M2-polarized macrophages and MDSCs while promoting phagocytosis and T-cell activation.
[54]	147	2026	Quantitative detection of macrophage activation in	Osteosarcoma	Orthotopic + Xenograft + Syngeneic	Human cell line	Both	Moderate (human cells in immunodeficient host)	Monotherapy / not applicable	Twenty female BALB/c mice with intratibial murine K7M2 osteosarcomas, twenty female

			osteosarcoma comparing 18F-FDG PET/CT with fe		＋Cell line					NOD scid gamma (NSG) mice with intratibial human 143B tumors, and twenty male NSG mice with intratibial human MG63.3 tumors were treated with either phosphate-buffered saline (PBS) or murine/human CD47 mAb (n = 10 per arm) and underwent either 18F-FDG PET/CT or ferumoxytol-enhanced MRI (n = 5 per group).
[58]	148	2026	Tumor size limits macrophage activation and imaging response to CD47 blockade in osteosarco	Osteosarcoma	Orthotopic＋Xenograft＋Syngeneic＋Cell line	Human cell line	Both	Moderate (human cells in immunodeficient host)	Second immune checkpoint／Nanocarrier／Targeted inhibitor (STAT3 / kinase)	Daldrup-Link, Nanoparticle enhanced MRI can monitor macrophage response to CD47 mAb immunotherapy in osteosarcoma,

			ma.							Cell.
[73]	149	2025	STAT3 inhibition in combination with CD47 blockade inhibits osteosarcoma lung metastasis.	Osteosarcoma	Orthotopic＋Syngeneic＋Cell line	Human cell line	Both	Moderate (murine, immunocompetent)	Targeted inhibitor (STAT3 / kinase)	The therapeutic effect of the STAT3 inhibitor WP1066 was significantly increased when combined with anti-CD47, and was associated with increased activated CD8+ T cells, NK cells and macrophages in the lungs and lymph nodes.
[59]	150	2025	Engineered macrophage nanoparticles enhance microwave ablation efficacy in osteosarcoma via targ	Osteosarcoma	Orthotopic＋Xenograft＋Syngeneic＋Cell line	Human cell line	Immunocompetent	Moderate (human cells in immunodeficient host)	Radiotherapy / ablation／Nanocarrier	Genetically engineered M1-polarized macrophage membranes overexpressing SIRPalpha, coating microwave-responsive nano-Prussian blue particles (SIRPalpha-M@nanoPB), targeted tumor

										CD47, disrupted the don't-eat-me signal and restored M1 macrophage phagocytosis.
[24]	152	2024	CD47 in Osteosarcoma: Correlation with Metastasis and Macrophage-Mediated Phagocytosis.	Osteosarcoma／Liposarcoma／UPS/MFH	Orthotopic＋Xenograft＋Cell line	Human cell line	Immunodeficient	Moderate (human cells in immunodeficient host)	Monotherapy / not applicable	Although some patients with undifferentiated pleomorphic sarcomas and liposarcomas show positive responses to these inhibitors, most patients with sarcomas do not exhibit any objective response to immune checkpoint inhibitors [11–13].
[60]	153	2024	Novel Clinically Translatable Iron Oxide Nanoparticle for Monitoring Anti-CD47	Osteosarcoma	Orthotopic＋Xenograft＋Syngeneic＋Cell line	Human cell line	Both	Moderate (human cells in immunodeficient host)	Nanocarrier	Children and young adults with metastatic osteosarcomas have a dismal 5-year survival rate of 30% or less.1 Therefore, new therapeutic

			Cancer Immunother						strategies are needed.
[72]	154	2024	Circumventing resistance within the Ewing sarcoma microenvironment by combinatorial innate immun	Ewing sarcoma	Orthotopic＋Xenograft＋Cell line	Patient-derived	Immuno deficient	High (patient-derived / humanized)	CAR-T / engineered cellular therapy	Conclusions Our preclinical studies demonstrate that immunotherapy via the innate immune system by combining tumor-targeting CAR-NK cells with an IL-15 agonist and a CD47 blockade is a promising novel therapeutic approach to targeting MCAMhigh malignant metastatic ES.
[67]	155	2024	Combinatorial macrophage induced innate immunotherapy against	Ewing sarcoma	Humanized＋Orthotopic＋Xenograft＋Syngeneic	Patient-derived	Immuno deficient	High (patient-derived / humanized)	Chemotherapy／Calreticulin / ICD induction	Here, we investigate the combination of a CD47 blockade (magrolimab, MAG) to inhibit the anti-phagocytic signal and a

			Ewing sarcoma: Turning "Two Keys"		＋Cell line					chemotherapy regimen (doxorubicin, DOX) to enhance the pro-phagocytic signal to induce macrophage phagocytosis of ES cells in vitro and inhibit tumor growth and metastasis in vivo.
[61]	156	2024	Codelivery of anti-CD47 antibody and chlorin e6 using a dual pH-sensitive nanodrug for photodyna	Osteosarcoma	Xenograft ＋ Syngeneic ＋Cell line	Human cell line	Immunocompetent	Moderate (human cells in immunodeficient host)	Radiotherapy / ablation／Nanocarrier／Calreticulin / ICD induction	Moreover, both calreticulin released during ICD and CD47 blockade can accelerate phagocytosis of tumor cells by macrophages, promote antigen presentation, and eventually induce T lymphocyte-mediated antitumor immunity.

[31]	158	2024	YB-1-based oncolytic virotherapy in combination with CD47 blockade enhances phagocytosis of pedi	Unspecified/pan-sarcoma	Xenograft ＋Cell line	Human cell line	Immuno deficient	Moderate (human cells in immunodeficient host)	Oncolytic virus ／Calreticulin / ICD induction	(2) XVir-infection enhances phagocytosis in general and (3) the combination of XVir and CD47i compared to controls showed by far superior enhancement of phagocytosis, tumor cell killing and innate immune activation.
[68]	160	2024	Engagement of CD99 Activates Distinct Programs in Ewing Sarcoma and Macrophages.	Osteosarcoma ／Ewing sarcoma	Orthotopic＋ Xenograft ＋Cell line	Patient-derived	Immuno deficient	High (patient-derived / humanized)	Calreticulin / ICD induction	Treating patients with Ewing sarcoma (EWS), the second most frequent pediatric tumor of the bone, remains a challenge for oncologists because of the paucity of novel effective drugs for patients who do not respond to first-line

										chemotherapy and for patients with metastatic disease.
[74]	162	2023	LPS combined with CD47mAb enhances the anti-osteosarcoma ability of macrophages.	Osteosarcoma	Cell line	Human cell line	Immunocompetent	Low (unspecified in vivo)	Innate immune agonist (LPS / TLR)	Furthermore, cell proliferation analysis, cell migration assay and apoptosis determination confirmed LPS-polarized macrophages might efficiently suppress OS cells growth and migration while promoting apoptosis.
[62]	163	2023	A nanodrug combining CD47 and sonodynamic therapy efficiently inhibits osteosarcoma	Osteosarcoma	Orthotopic＋Syngeneic＋Cell line	Human cell line	Immunocompetent	Moderate (murine, immunocompetent)	Radiotherapy / ablation／Nanocarrier／Calreticulin / ICD induction	A biomimetic nanodrug (MPIRx) co-loading the sonosensitizer IR780 and the CD47 inhibitor RRx-001 promoted M1 polarization and macrophage phagocytosis, inhibiting

			deterioratio							subcutaneous and orthotopic osteosarcoma and pulmonary metastasis.
[55]	164	2023	A humanized orthotopic mouse model for preclinical evaluation of immunotherapy in Ewing sarcoma.	Ewing sarcoma	Humanized＋Orthotopic＋Xenograft＋Syngeneic＋Cell line	Patient-derived	Immuno deficient	High (patient-derived / humanized)	Monotherapy / not applicable	Treatment with magrolimab induces CD47 blockade resulting in significantly decreased primary tumor growth, decreased lung metastasis and prolonged animal survival in the established humanized model.
[25]	165	2023	Expression and Clinical Significance of Various Checkpoint Molecules in Advanced Osteosarco	Osteosarcoma	Xenograft＋Cell line	Human cell line	Immuno deficient	Moderate (human cells in immunodeficient host)	Monotherapy / not applicable	Associations between the immunohischemistry markers and clinicopathological variables and survival were evaluated by the $\chi 2$ displayed by cross-table, Cox proportional

			ma: P							hazards regression model, and Kaplan–Meier plots.
[29]	168	2022	Metabolic control of CD47 expression through LAT2-mediated amino acid uptake promotes tumor immu	Osteosarcoma	Xenograft ＋Cell line	Human cell line	Immuno deficient	Moderate (human cells in immunodeficient host)	Chemotherapy	We reveal that macrophages in response to chemotherapy secrete interleukin-18, which in turn upregulates expression of L-amino acid transporter 2 (LAT2) in tumor cells for substantially enhanced uptakes of leucine and glutamine, two potent stimulators of mTORC1.
[30]	169	2022	Anti-GD2 synergizes with CD47 blockade to mediate tumor	Osteosarcoma	Orthotopic＋ Xenograft ＋ Syngeneic ＋Cell line	Human cell line	Immuno deficient	Moderate (human cells in immunodeficient host)	Second immune checkpoint	Anti-GD2 antibodies have improved survival rates for patients with neuroblastoma only when

			eradication.							administered as part of intense chemotherapy-based cytotoxic regimens7–9, which are associated with debilitating late effects, including hearing loss, growth retardation and secondary leukemias10,11.
[70]	170	2021	Engineering Oncolytic Vaccinia Virus to redirect Macrophages to Tumor Cells.	Osteosarcoma	Xenograft ＋ Syngeneic ＋Cell line	Human cell line	Immunocompetent	Moderate (human cells in immunodeficient host)	Second immune checkpoint／Oncolytic virus	Lack of antitumor activity is most likely due to limited viral spread through the tumor and the immunosuppressive tumor microenvironment.
[20]	172	2020	Tumor-associated macrophages and macrophage-related immune	Liposarcoma／UPS/MFH／Angiosarcoma	Xenograft	Murine	Immunodeficient	Low (unspecified in vivo)	Monotherapy / not applicable	To enhance our understanding of the sarcoma immune landscape, we quantified and characterized

			checkpoint expression in sarcomas.							tumor-associated macrophage infiltration and expression of the targetable macrophage-related immune checkpoint CD47/SIRPα across sarcoma types.
[69]	174	2019	Improving the efficacy of osteosarcoma therapy: combining drugs that turn cancer cell 'don't eat	Osteosarcoma	Orthotopic＋Xenograft＋Cell line	Human cell line	Both	Moderate (human cells in immunodeficient host)	Chemotherapy	The long-term survival of osteosarcoma patients with metastatic or recurrent disease remains dismal, and new therapeutic options are urgently needed.
[43]	175	2019	Nanoparticle enhanced MRI can monitor macrophage response	Osteosarcoma	Orthotopic＋Xenograft＋Syngeneic＋Cell line	Human cell line	Both	Moderate (human cells in immunodeficient host)	Nanocarrier	Compared to IgG-treated controls, CD47 mAb-treated tumors demonstrated significantly shortened T2

			to CD47 mAb immunotherapy in osteosarc						relaxation times on ferumoxytol-MRI scans ($p < 0.01$) and significantly increased F4/80+CD80+ M1 macrophages on histopathology ($p < 0.01$).
[44]	176	2019	Anti-CD47 antibody eliminates bone tumors in rats.	Osteosarcoma	Cell line	Human cell line	Immunocompetent	Low (unspecified in vivo)	Monotherapy / not applicable	Taken together, the results of this study suggested that CD47 is a key regulator of bone tumor cell metastasis and that targeting inhibition of anti-CD47 may be a new immunotherapy for bone tumors.
[28]	177	2015	CD47 blockade inhibits tumor progression human osteosarco	Osteosarcoma	Xenograft ＋Cell line	Patient-derived	Immuno deficient	High (patient-derived / humanized)	Monotherapy / not applicable	Finally, CD47 blockade increases macrophage phagocytosis of osteosarcoma tumor cells. In conclusion, our

			ma in xenograft models.							findings demonstrate that CD47 is a critical regulator in the metastasis of osteosarcoma and suggest that targeted inhibition of this antigen by anti-CD47 may be a novel immunotherapeutic approach in the management of this tumor.
[23]	178	2016	Abstract 2471: Evaluation of CD47 expression and effects of CD47-SIRPα fusion protein in patient	Osteosarcoma	Xenograft	Patient-derived	Immuno deficient	High (patient-derived / humanized)	Monotherapy / not applicable	CD47 is a commonly expressed trans-membrane protein which binds to signal regulatory protein α (SIRPα) expressed on the surface of the macrophages resulting in inhibition of phagocytosis and cell clearance.

[66]	180	2014	CD47 in the tumor microenvironment limits cooperation between antitumor T-cell immunity and radi	Fibrosarcoma	Xenograft ＋ Syngeneic ＋Cell line	Human cell line	Immunocompetent	Moderate (human cells in immunodeficient host)	Radiotherapy / ablation	Mechanistic investigations revealed increased tumor infiltration by cytotoxic CD8+ T cells in a CD47-deficient microenvironment, with an associated increase in T cell–dependent intratumoral expression of granzyme B. Correspondingly, an inverse correlation between CD8+ T-cell infiltration and CD47 expression was observed in human melanomas.
[63]	181	APR 2021	Fullerenols boosting the therapeutic effect of anti-CD47 antibody to	Osteosarcoma	Orthotopic＋ Xenograft ＋ Syngeneic ＋Cell line	Human cell line	Immunocompetent	Moderate (human cells in immunodeficient host)	Nanocarrier／Calreticulin / ICD induction	Moreover, the combined therapy shows significantly increased anti-tumor efficiency compared with

			trigger robust anti-tumor i							anti-CD47 mAb alone in both U87MG subcutaneous glioblastoma model and 143B orthotopic osteosarcoma model.
[64]	182	OCT 15 2024	Immune checkpoint-regulatable hydrogel-immobilized nanotherapeutics for post-surgical treatment	Osteosarcoma	Orthotopic＋Cell line	Human cell line	NR	Low (unspecified in vivo)	Chemotherapy ／Nanocarrier ／Calreticulin / ICD induction	In this study, we report the fabrication of an in-situ injectable hydrogel-immobilized nanotherapeutics, tailored to stimulate tumoricidal immunity, thus mitigate both localized osteosarcoma recurrence and the propagation of metastatic tumors subsequent to surgical intervention.

Table S9. Interventional trials enrolling sarcoma patients. Interventional trials of CD47–SIRPα-targeted agents enrolling sarcoma patients (n = 2). Sarcoma enrollment is reported separately because both trials also enrolled non-sarcoma cohorts. ORR, objective response rate; PFS, progression-free survival; TRAE, treatment-related adverse event. Reference numbers follow the numbering of the main text; studies are ordered by sarcoma subtype and, within subtype, by cohort rather than by reference number.

Trial	Agent	Phase	Sarcoma Enrollment	Status	Results Posted
NCT04751383 [33]	magrolimab + dinutuximab	1	12 (with neuroblastoma; sarcoma-specific n not reported separately)	Terminated	No
NCT04996004 [15]	ontorpacept + doxorubicin	1/2	76 (high-grade leiomyosarcoma)	Completed	Yes

Table S10. Patient-cohort and translational studies using human sarcoma material. Patient-cohort and translational studies using human sarcoma material (n = 16). N is the number of sarcoma patients or specimens analyzed (patient count where both were reported). Assay details are given where stated by the source publication. IHC, immunohistochemistry; TMA, tissue microarray; FFPE, formalin-fixed paraffin-embedded; TAM, tumor-associated macrophage. Reference numbers follow the numbering of the main text; studies are ordered by sarcoma subtype and, within subtype, by cohort rather than by reference number.

Ref	Study ID	Year	Subtype	Sample Source	N	Assay	Clinical Variable	Key Finding
[35]	41	2023	Soft-tissue sarcoma (unspecified)	Serum	152	Serum macrophage biomarkers (sCD163, sCD206, sSIRPα, sLILRB1)	Mortality	sCD163 and sSIRPα predicted mortality (prospectively collected clinical data)

[22]	166		Soft-tissue sarcoma (multiple)	Resected tumor tissue (ex vivo TIIC culture)	66	CD47 IHC + ex vivo anti-CD47	Ex vivo response profile	CD47 highly expressed in most STS; strongest anti-CD47 response in UPS, strongest anti-PD-1 response in high-grade LMS
[50]	60	2022	Sarcoma (pediatric)	Patient monocytes + HuO9-H3 cell line	not reported	Anti-CD47 blocking assay	Phagocytosis	Anti-CD47 increased phagocytosis (53.16% vs 12.06%, p = 0.0456)
[42]	128	2014	Ewing sarcoma; osteosarcoma	Xenograft models	not reported	Humanised anti-CD47 mAb	CD47 expression; xenograft efficacy	CD47 expressed on Ewing sarcoma; antitumour efficacy in osteosarcoma xenografts
[26]	130	2026	Leiomyosarcoma (ST-LMS 23; U-LMS 32)	Tissue microarray	55	Spatial RNA–protein profiling (GeoMx DSP)	Overall survival	High CD47 associated with improved survival in ST-LMS (HR 0.76, 95% CI 0.08–0.88, p = 0.021)
[45]	133	2025	Osteosarcoma	Plasma + tumor tissue	67	Soluble checkpoint panel	Risk stratification	Soluble checkpoint factors identify high-risk osteosarcoma subtypes

[47]	141	2020	Chondrosarcoma (27 conventional; 49 dedifferentiated)	FFPE tissue	49	IHC (CD3/CD8/CD68/CD163) + RT-qPCR ICP panel	Prognosis; immune landscape	Immunosuppressive environment; TAM-dominant infiltrate; PD-L1 in 42.6% of dedifferentiated CHS
[15]	144	2026	Leiomyosarcoma	Trial patients	76	Trial outcomes (see Table S9)	Efficacy; safety	See Table S9
[24]	152	2024	Osteosarcoma	Diagnostic biopsies	24	CD47 IHC	Metastasis	CD47 positivity correlated with clinicopathological features; 5/24 (20.8%) positive
[27]	159	2024	Myeloid sarcoma	Bone marrow biopsies	84	CD47 and SIRPα IHC	Prognosis	CD47 expression a favorable prognostic factor; ~80% negative for nSIRPα
[25]	165	2023	Osteosarcoma (heavily pre-treated)	Tumor tissue	35	Multi-checkpoint IHC	Checkpoint co-expression	CD47 positive in 5/35 (14.3%); diverse staining intensities
[29]	168	2022	Osteosarcoma	Matched pre/post-chemotherapy tissue	81	CD47 H-score	Chemotherapy-induced CD47 upregulation	CD47 upregulated after chemotherapy and associated with patient mortality
[20]	172	2020	Multiple (24–27 types)	Tissue microarray (1,242	1242	CD47 & SIRPα IHC (B6H12)	Prognosis; subtype	See Table 1 — bimodal expression; 47.5% at least focal;

				specimens)			distribution	CD47 adverse in osteosarcoma
[43]	175	2019	Osteosarcoma	Patient specimens + osteoma/normal bone	8	Ferumoxytol-enhanced MRI	Macrophage response imaging	CD47 protein higher in osteosarcoma than osteoma or normal bone; feasibility of imaging macrophage response
[28]	177	2015	Osteosarcoma	Freshly isolated tumor tissue + 30-patient cohort	30	CD47 mRNA	Prognosis	CD47 mRNA up-regulated in osteosarcoma; high CD47 mRNA associated with outcome in a 30-patient cohort
[23]	178	2016	Osteosarcoma	Tissue microarray (81 specimens)	81	Anti-CD47 (B6H12) IHC	Prevalence; prognosis	CD47 expressed in 87.7% of specimens
